# Hyperfine spectroscopy in a quantum-limited spectrometer

**DOI:** 10.5194/mr-1-315-2020

**Published:** 2020-12-17

**Authors:** Sebastian Probst, Gengli Zhang, Miloš Rančić, Vishal Ranjan, Marianne Le Dantec, Zhonghan Zhang, Bartolo Albanese, Andrin Doll, Ren Bao Liu, John Morton, Thierry Chanelière, Philippe Goldner, Denis Vion, Daniel Esteve, Patrice Bertet

**Affiliations:** 1 Quantronics group, SPEC, CEA, CNRS, Université Paris-Saclay, CEA Saclay, 91191 Gif-sur-Yvette CEDEX, France; 2 Department of Physics and The Hong Kong Institute of Quantum Information Science and Technology, The Chinese University of Hong Kong, Shatin, New Territories, Hong Kong, China; 3 Chimie ParisTech, PSL University, CNRS, Institut de Recherche de Chimie Paris, 75005 Paris, France; 4 Laboratory of nanomagnetism and oxides, SPEC, CEA, CNRS, Université Paris-Saclay, CEA Saclay, 91191 Gif-sur-Yvette CEDEX, France; 5 London Centre for Nanotechnology, University College London, London WC1H 0AH, UK; 6 Univ. Grenoble Alpes, CNRS, Grenoble INP, Institut Néel, 38000 Grenoble, France

## Abstract

We report measurements of electron-spin-echo envelope modulation (ESEEM) performed at millikelvin temperatures in a custom-built high-sensitivity spectrometer based on superconducting micro-resonators. The high quality factor and small mode volume (down to 0.2 pL) of the resonator allow us to probe a small number of spins, down to $5\times 10^{2}$ . We measure two-pulse ESEEM on two systems: erbium ions coupled to ${}^{183}W$ nuclei in a natural-abundance ${\mathrm{CaWO}}_{4}$ crystal and bismuth donors coupled to residual ${}^{29}\mathrm{Si}$ nuclei in a silicon substrate that was isotopically enriched in the ${}^{28}\mathrm{Si}$ isotope. We also measure three- and five-pulse ESEEM for the bismuth donors in silicon. Quantitative agreement is obtained for both the hyperfine coupling strength of proximal nuclei and the nuclear-spin concentration.

## 1 Introduction

Electron paramagnetic resonance (EPR) spectroscopy provides a set of versatile tools to study the magnetic environment of unpaired electron spins [Bibr bib1.bibx44]. EPR spectrometers rely on the inductive detection of the spin signal by a three-dimensional microwave resonator tuned to the spin Larmor frequency. While concentration sensitivity is the main concern for dilute samples available in macroscopic volumes [Bibr bib1.bibx48], there are also cases in which the absolute spin detection sensitivity matters, motivating research towards alternative detection methods to measure smaller and smaller numbers of spins. Electrical [Bibr bib1.bibx14], optical [Bibr bib1.bibx53], and scanning-probe-based [Bibr bib1.bibx42] detection methods of magnetic resonance have reached sufficient sensitivity to detect individual electron spins.

In parallel, recent results have shown that the inductive detection method can also be pushed to much higher absolute sensitivity than previously achieved, using planar micro-resonators [Bibr bib1.bibx31] and micro-helices [Bibr bib1.bibx45]. Superconducting resonators [Bibr bib1.bibx50] are particularly useful in that context since they combine low mode volume and narrow linewidth κ . Inductive-detection spectrometers relying on a superconducting planar micro-resonator combined with a Josephson parametric amplifier (JPA), cooled down to millikelvin temperatures [Bibr bib1.bibx7], have achieved a sensitivity of 10 spin $/\sqrt{\mathrm{Hz}}$ for detecting Hahn echoes emitted by donors in silicon [Bibr bib1.bibx40]. A particular feature of these quantum-limited spectrometers is that quantum fluctuations of the microwave field play an important role. First, the system output noise is governed by these quantum fluctuations, with negligible thermal noise contribution. Second, quantum fluctuations also impact spin dynamics by triggering spontaneous emission of microwave photons at a rate $\Gamma_{P}=4g^{2}/\kappa$ , g being the spin–photon coupling [Bibr bib1.bibx8]. This Purcell effect forbids $T_{1}$ from becoming prohibitively long since it is at most equal to $\Gamma_{P}^{-1}$ , making spin detection with a reasonable repetition rate possible even at the lowest temperatures.

Hahn echoes are the simplest pulse sequence used in EPR spectroscopy, useful to determine the electron-spin density as well as the spin Hamiltonian parameters and their distribution. The richness of EPR comes from the ability to characterize the local magnetic environment of the electron spins, often consisting of a set of nuclear spins or of other electron spins. For that, hyperfine spectroscopy is required, which uses more elaborate pulse sequences and requires larger detection bandwidth. Previous hyperfine spectroscopy measurements with superconducting micro-resonators include the electron–nuclear double resonance detection of donors in silicon [Bibr bib1.bibx47] and the electron-spin-echo envelope modulation (ESEEM) of erbium ions by the nuclear spin of yttrium in a $Y_{2}{\mathrm{SiO}}_{5}$ crystal [Bibr bib1.bibx35].

Here, we demonstrate that hyperfine spectroscopy is compatible with quantum-limited EPR spectroscopy despite its additional requirements in terms of pulse complexity and bandwidth, by measuring ESEEM in two model electron-spin systems. We measure the ESEEM of erbium ions coupled to ${}^{183}W$ nuclei in a scheelite crystal ( ${\mathrm{CaWO}}_{4}$ ) with a simple two-pulse sequence and get quantitative agreement with a simple dipolar interaction model. We also measure the ESEEM of bismuth donors in silicon caused by ${}^{29}\mathrm{Si}$ nuclei using two-, three-, and five-pulse sequences [Bibr bib1.bibx44]. Compared to other ESEEM measurements on donors in silicon [Bibr bib1.bibx51], ours are performed in an isotopically purified sample having a 100 times lower concentration in ${}^{29}\mathrm{Si}$ ( 500 ppm) than natural abundance. As a result, the dominant hyperfine interactions in the ESEEM signal are very low (on the order of 100 Hz) and have to be detected at low magnetic fields (around 0.1 mT). These results bring quantum-limited EPR spectroscopy one step closer to real-world applications.

## 2 ESEEM spectroscopy: theory

### 2.1 Phenomenology

We start by briefly discussing the ESEEM phenomenon. Consider an ensemble of electron spins placed in a magnetic field $B_{0}$ . The spin ensemble linewidth Γ is broadened by a variety of mechanisms: spatial inhomogeneity of the applied field $B_{0}$ , local magnetic fields generated by magnetic impurities throughout the sample, and spatially inhomogeneous strain or electric fields. One prominent way to mitigate the effect of this inhomogeneous broadening is the spin-echo sequence (also called Hahn echo, or two-pulse echo). It consists of a π/2 pulse at time t=0 and a π pulse after a delay τ (see Fig. [Fig Ch1.F1]a). This π pulse reverses the evolution of the phase of the precessing magnetic dipoles, which leads at a later time 2τ to their refocusing and the emission of a microwave pulse (the echo) of amplitude $V_{2p}(\tau )$ .

**Figure 1 Ch1.F1:**
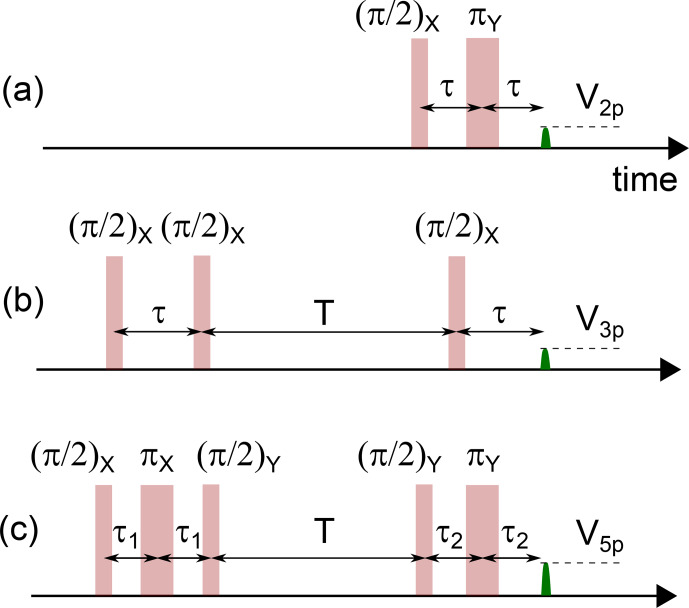
Sequences used for two-pulse **(a)**, three-pulse **(b)**, and five-pulse **(c)** ESEEM measurements.

In general, $V_{2p}(\tau )$ decays monotonically; it can however also display oscillations. Such ESEEM was first observed by [Bibr bib1.bibx27] and [Bibr bib1.bibx41] for ${\mathrm{Ce}}^{3+}$ ions in a ${\mathrm{CaWO}}_{4}$ crystal and was interpreted as being caused by the dipolar interaction of the electronic spin of the ${\mathrm{Ce}}^{3+}$ ions with the ${}^{183}W$ nuclear spins of the crystal. The oscillation frequencies appearing in the ESEEM pattern are related to the nuclear-spin Larmor frequencies and to their coupling to the electron spin. As such, ESEEM measurements provide spectroscopic information on the nature of the nuclear-spin bath and its density, and ESEEM spectroscopy has become an essential tool in advanced EPR [Bibr bib1.bibx44]. ESEEM has also been observed for individual spins measured optically, in particular for individual nitrogen vacancy (NV) centers in diamond coupled to a bath of ${}^{13}C$ nuclear spins [Bibr bib1.bibx11]. A more complete theory of ESEEM is presented in [Bibr bib1.bibx26]. Our goal here is to provide a simple picture of the physics involved, as well as to introduce useful formulas and notations.

### 2.2 Two-spin 1/2 model

We follow the analysis in [Bibr bib1.bibx44] of the model case depicted in Fig. [Fig Ch1.F2]a. An electron spin of S=1/2 , with an isotropic g tensor, is coupled to a proximal nuclear spin of I=1/2 . Both are subject to a magnetic field $B_{0}$ applied along z . The system Hamiltonian is

1 $$H_{0}=H_{e}+H_{n}+H_{\mathrm{hf}},$$

where $H_{e}=\omega_{S}S_{z}$ ( $H_{n}=\omega_{I}I_{z}$ ) is the Zeeman Hamiltonian of the electron (nuclear) spin with Larmor frequency $\omega_{S}$ ( $\omega_{I}$ ), and $H_{\mathrm{hf}}$ is the electron–nuclear hyperfine interaction, which includes their dipole–dipole coupling and may include a Fermi contact term as well. We assume that $\omega_{S}$ is much larger than the hyperfine interaction strength, in which case terms proportional to the $S_{x}$ and $S_{y}$ operators can be neglected. This secular approximation leads to a hyperfine Hamiltonian of the form $H_{\mathrm{hf}}=AS_{z}I_{z}+BS_{z}I_{x}$ , with the expressions for A and B depending on the details of the hyperfine interaction [Bibr bib1.bibx44].

Overall, the system Hamiltonian is

2 $$H_{0}=\omega_{S}S_{z}+\omega_{I}I_{z}+AS_{z}I_{z}+BS_{z}I_{x}.$$

**Figure 2 Ch1.F2:**
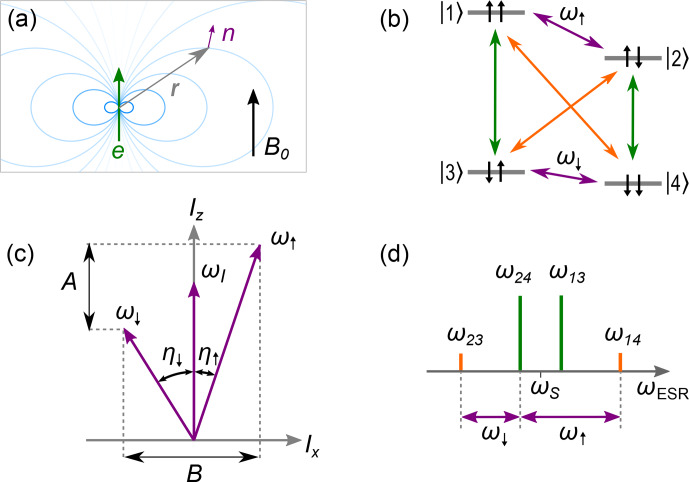
ESEEM model system for an electron spin of S=1/2 and nuclear spin of I=1/2 with $\omega_{I},A,B>0$ . **(a)** Nuclear spin (purple) subject to external field $B_{0}$ and dipole field (blue) of a nearby electron spin (green) located at relative position r . **(b)** Energy diagram showing the electron transitions (green), the nuclear transitions (purple), and the (normally forbidden) electro-nuclear transitions (orange). The energy levels |1〉,…,|4〉 are labeled according to the eigenstates of the Zeeman basis. **(c)** Quantization axes $\omega_{↑}$ and $\omega_{↓}$ due to mixing of the nuclear states, which results in inclination of the quantization axis from z by the angles $\eta_{↑}$ and $\eta_{↓}$ , respectively. **(d)** EPR spectrum showing the electron transitions (green) and the electro-nuclear transitions (orange) as well as the relation of these electron spin resonance (ESR) transitions to the nuclear frequencies $\omega_{↑}$ and $\omega_{↓}$ (purple).

Because of the $BS_{z}I_{x}$ term, the nuclear spin is subjected to an effective magnetic field whose direction (and magnitude) depend on the electron-spin state $\left|{↑}_{e}\rangle \right.$ or $\left|{↓}_{e}\rangle \right.$ . Its eigenstates therefore depend on the electron-spin state, so that transitions become allowed between all the spin system energy levels $\left|1\rangle -\right|4\rangle$ , leading to the ESEEM phenomenon. Relevant parameters are the electron-spin-state-dependent angles between the effective magnetic field seen by the nuclear spin and the quantization axis z :

3 $$\begin{array}{rl} & \eta_{↑}=\mathrm{arctan}\frac{B}{A+2\omega_{I}}\\ & \eta_{↓}=\mathrm{arctan}\frac{B}{A-2\omega_{I}}, \end{array}$$

and the electron-spin-dependent nuclear-spin frequencies:

$$\begin{array}{cc} \omega_{↑} & =\left(\omega_{I}+\frac{A}{2}\right)\mathrm{cos}\eta_{↑}-\frac{B}{2}\mathrm{sin}\eta_{↑}\\ \omega_{↓} & =\left(\omega_{I}-\frac{A}{2}\right)\mathrm{cos}\eta_{↓}-\frac{B}{2}\mathrm{sin}\eta_{↓}. \end{array}$$

When $\eta_{↑}$ , $\eta_{↓}$ are close to equal, only the nuclear-spin-preserving transitions are allowed; this occurs either when B=0 (due to a specific orientation of the dipolar field or a purely isotropic hyperfine coupling), or when B≠0 but $\omega_{I}\gg A$ (very weak-coupling limit) or $\omega_{I}\ll A$ (very strong coupling limit). On the contrary, when the direction of the effective magnetic field seen by the nuclear spin is electron-spin dependent, all transitions become allowed. This occurs when B≠0 and $\omega_{I}≃\pm A/2$ .

### 2.3 Multi-pulse ESEEM

Because of the level structure shown in Fig. [Fig Ch1.F2], and assuming for simplicity microwave pulses so short that their bandwidth is much larger than $\omega_{↑,↓}$ , microwave pulses at the electron-spin frequency $\omega_{S}$ excite the allowed transitions |1〉↔|3〉 and |2〉↔|4〉 but also the normally forbidden |1〉↔|4〉 and |2〉↔|3〉 , leading to coherence transfer between the levels and to beatings. Note that for simplicity we assume that the microwave pulses are ideal and so short that their bandwidth is much larger than $\omega_{12}$ and $\omega_{34}$ .

It is then possible to compute analytically the effect of a two-pulse echo sequence consisting of an instantaneous ideal π/2 pulse and an instantaneous ideal π pulse (see Fig. [Fig Ch1.F1]), disregarding any decoherence. The resulting echo amplitude [Bibr bib1.bibx44] is given by

4 $$\begin{array}{rl} V_{2p}(\tau ) & =1-\frac{k}{4}[2-2\mathrm{cos}\left(\omega_{↑}\tau \right)-2\mathrm{cos}\left(\omega_{↓}\tau \right)\\ & \;+\mathrm{cos}\left(\left(\omega_{↑}-\omega_{↓}\right)\tau \right)+\mathrm{cos}\left(\left(\omega_{↑}+\omega_{↓}\right)\tau \right)], \end{array}$$

with

5 $$k={\left[\frac{B\omega_{I}}{\omega_{↑}\omega_{↓}}\right]}^{2}.$$

The spin-echo amplitude is modulated by a function whose frequency spectrum and amplitude contain information about the nuclear-spin Larmor frequency $\omega_{I}$ as well as its hyperfine coupling (A,B) to the electron spin. The modulation contrast 0≤k≤1 is maximal when transitions |1〉-|4〉 and |2〉-|3〉 are maximally allowed, corresponding to $\omega_{I}≃A/2$ .

The above results are exact, as long as the secular approximation is valid and the pulses are ideal. In the weak-coupling limit A , $B\ll \omega_{I}$ , $\omega_{↑}≃\omega_{↓}≃\omega_{I}$ so that $V_{2p}(\tau )=1-\frac{k}{4}[3-4\mathrm{cos}(\omega_{I}\tau )+\mathrm{cos}(2\omega_{I}\tau )]$ , with $k=(B/\omega_{I}{)}^{2}\ll 1$ . In this limit, the echo modulation spectrum directly yields the nuclear-spin Larmor frequency and also contains components at twice this frequency. Note however that, in practice, the π pulse bandwidth is always finite, because of the resonator bandwidth or limited pulse power; this sets a limit to the range of detectable modulation frequencies.

The electron spin is often coupled to N nuclear spins, with N>1 . Since all nuclear-spin subspaces can be diagonalized separately, the total ESEEM modulation is simply given by the product of each nuclear-spin modulation $V_{2p,\;l}(\tau )$ , l being the nuclear-spin index. Taking also into account that the electron spin is also subject to decoherence processes, modeled, for instance, by an exponential decay with time constant $T_{2}$ , the echo envelope is

6 $$V_{2p}^{\prime }(\tau )=\mathrm{exp}(-2\tau /T_{2})\prod_{l=1}^{N}V_{2p,\;l}(\tau ).$$

The modulation pattern $V_{2p}^{\prime }(\tau )$ yields quantitative information about the nature and coupling of the nuclear spins surrounding the electron spin whose echo is measured and is therefore a useful tool in EPR spectroscopy. When the environmental nuclei have a certain probability p to be of a given isotope with a nuclear spin of I=1/2 , and a probability 1-p to be of an isotope with I=0 , the above formulas are straightforwardly modified [Bibr bib1.bibx41] by writing

7 $$\begin{array}{rl} V_{2p,\;l}(\tau ) & =1-\frac{pk_{l}}{4}[2-2\mathrm{cos}\left(\omega_{↑,l}\tau \right)-2\mathrm{cos}\left(\omega_{↓,l}\tau \right)\\ & \;+\mathrm{cos}\left(\left(\omega_{↑,l}-\omega_{↓,l}\right)\tau \right)+\mathrm{cos}\left(\left(\omega_{↑,l}+\omega_{↓,l}\right)\tau \right)]. \end{array}$$

The echo signal $V_{2p}^{\prime }(\tau )$ is the sum of terms that have the general form $p^{L}\prod_{l=1}^{l=L}k_{l}\mathrm{cos}(\omega_{\mu ,l}\tau )$ , where l runs over a subset of L nuclei and μ=↑,↓ . If p≪1 , this expression is well approximated by keeping only the L=1 terms, which then yields

8 $$\begin{array}{rl} V_{2p}(\tau ) & ≃1-\sum_{l=1}^{l=N}\frac{pk_{l}}{4}[2-2\mathrm{cos}\left(\omega_{↑,l}\tau \right)-2\mathrm{cos}\left(\omega_{↓,l}\tau \right)\\ & \;+\mathrm{cos}\left(\left(\omega_{↑,l}-\omega_{↓,l}\right)\tau \right)+\mathrm{cos}\left(\left(\omega_{↑,l}+\omega_{↓,l}\right)\tau \right)]. \end{array}$$

One limitation of the previous pulse sequence is that the modulation envelope can only be measured up to a time of order $T_{2}$ due to electron-spin decoherence, which may be too short for appreciable spectral resolution. This limitation can be overcome by the three-pulse echo sequence shown in Fig. [Fig Ch1.F1]b. It consists of a π/2 pulse applied at t=0 followed, after a time τ chosen such that $\tau <T_{2}$ , by a second π/2 pulse. After a variable delay T , a third π/2 pulse is applied, leading to the emission of a stimulated echo at time t=T+2τ . The interest of this sequence is that the first pair of π/2 pulses generates nuclear-spin coherence that can survive up to the nuclear-spin coherence time $T_{2,\;n}$ which is in general much longer than $T_{2}$ (and close to the electron energy spin relaxation time $T_{1}$ ). An analytical formula can be derived for the three-pulse echo amplitude in the ideal pulse approximation [Bibr bib1.bibx44]:

9 $$\begin{array}{rl} V_{3p}(T) & =\mathrm{exp}(-T/T_{2,\;n})\mathrm{exp}(-2\tau /T_{2})\\ & \;\{1-\frac{k}{4}[\left[1-\mathrm{cos}\omega_{↓}\tau \right]\left[1-\mathrm{cos}\omega_{↑}(T+\tau )\right]\\ & \;+\left[1-\mathrm{cos}\omega_{↑}\tau \right]\left[1-\mathrm{cos}\omega_{↓}(T+\tau )\right]]\}. \end{array}$$

Contrary to two-pulse ESEEM, three-pulse echo modulation as a function of T only contains the $\omega_{↓},\omega_{↑}$ frequency components, and not their sum or difference; that is, in the weak-coupling limit A , $B\ll \omega_{I}$ , only the nuclear-spin Larmor frequency $\omega_{I}$ appears in the spectrum. Another difference is that the modulation pattern and amplitude depend on τ ; in particular, its amplitude is zero whenever $\omega_{↓,↑}\tau =2\pi n$ with n integer (“blind spots”).

For weakly coupled nuclei, the modulation amplitude of three-pulse ESEEM can be enhanced by up to 1 order of magnitude by using a more complex pulse sequence known as five-pulse ESEEM [Bibr bib1.bibx44] and shown in Fig. [Fig Ch1.F1]. The analytical formula for the five-pulse echo amplitude $V_{5p}$ is given in the Supplement.

Equation ([Disp-formula Ch1.E6]), with proper modification to take into account contributions of different pathways, can be applied to the three- and five-pulse ESEEM to treat coupling to multiple nuclear spins. The details are shown in Sect. S3.3 of the Supplement.

### 2.4 Fictitious spin model

The electronic spins that we consider in this work involve an unpaired electron with spin of $S_{0}=1/2$ either located around or trapped by an ionic defect, which itself can possess a non-zero nuclear spin of $I_{0}$ . These two spins of the defect are strongly coupled and therefore form a multi-level system, which can nevertheless be mapped to an effective, fictitious, spin- 1/2 model as explained below [Bibr bib1.bibx44], to which the model of Sect. 2.3 can be applied.

The system spin Hamiltonian writes

10 $$H_{\mathrm{ion}}=\beta_{e}B_{0}\cdot g_{e}\cdot S_{0}+S_{0}\cdot A_{0}\cdot I_{0}.$$

Here, $\beta_{e}$ is the electron Bohr magneton, $g_{e}$ is the (possibly anisotropic) gyromagnetic tensor, and $A_{0}$ the hyperfine tensor. The nuclear Zeeman interaction of the defect system, being small compared to the hyperfine interaction in the range of magnetic fields explored here, is neglected from the Hamiltonian.

This multi-level electron-spin system is coupled to other nuclear spins in the lattice, giving rise to ESEEM. Consider a nuclear spin at a lattice site j , defined by its location $r_{j}$ with respect to the electron spin. The nuclear Zeeman Hamiltonian is $H_{j}=\omega_{I}I_{j,z}$ , with $\omega_{I}=g_{n}\beta_{n}B_{0}$ , $g_{n}$ being the nuclear g factor and $\beta_{n}$ the nuclear magneton. Its hyperfine coupling to the electron-spin system is described by the Hamiltonian:

11 $$H_{j,\;\mathrm{hf}}=S_{0}\cdot A_{j}\cdot I_{j},$$

with

12 $$A_{j}=A_{j,\;\mathrm{cf}}+A_{j,\;\mathrm{dd}}.$$

This hyperfine tensor consists of a Fermi contact term, $A_{j,\;\mathrm{cf}}=\frac{2}{3}\;\mu_{0}\;\beta_{e}\;g_{n}\;\beta_{n}\;g_{e}{\left|\psi (r_{j})\right|}^{2}$ , and a dipole–dipole term, $A_{j,\;\mathrm{dd}}=\frac{3\mu_{0}}{4\pi {\left|r_{j}\right|}^{5}}\beta_{e}\;\beta_{n}\;g_{n}\left[r_{j}^{2}g_{e}-3\left(g_{e}\cdot r_{j}\right)r_{j}\right]$ , $\psi (r_{j})$ , being the electron wave function at the nuclear-spin location.

The Hamiltonian $H_{\mathrm{ion}}$ (Eq. [Disp-formula Ch1.E10]) can be diagonalized, yielding $4I_{0}+2$ energy levels. It is in general possible to isolate two levels |α〉 and |β〉 that are coupled by an electron spin resonance (ESR)-allowed transition and are resonant or quasi-resonant with the microwave cavity, with a transition frequency $\omega_{S}$ . If these two levels are sufficiently separated in energy from other levels of $H_{\mathrm{ion}}$ , they define a fictitious S=1/2 system. Writing the total Hamiltonian $H_{\mathrm{ion}}+H_{j}+H_{j,\;\mathrm{hf}}$ restricted to this two-dimensional subspace yields

13 $$\begin{array}{rl} H_{0} & =\omega_{S}S_{z}+\left(\omega_{I}+\frac{m_{S}^{\alpha }+m_{S}^{\beta }}{2}A_{j,zz}\right)I_{j,z}\\ & +\frac{m_{S}^{\alpha }+m_{S}^{\beta }}{2}A_{j,zx}I_{j,x}\\ & +\left(m_{S}^{\alpha }-m_{S}{}^{\beta }\right)\left(A_{j,zz}S_{z}I_{j,z}+A_{j,zx}S_{z}I_{j,x}\right)\;, \end{array}$$

where $m_{S}^{\alpha ,\;\beta }=\langle \alpha ,\beta |S_{0,z}|\alpha ,\beta \rangle$ .

Equation ([Disp-formula Ch1.E13]) maps the more complex system to the simple model of Sect. 2.2. Compared to Eq. ([Disp-formula Ch1.E2]), two differences appear. First, the hyperfine interaction parameters A , B are rescaled by the effective longitudinal magnetization difference $\left(m_{S}^{\alpha }-m_{S}^{\beta }\right)$ which depends on the two levels considered. Second, when the average longitudinal magnetization of the two levels $\left(m_{S}^{\alpha }+m_{S}^{\beta }\right)$ is non-zero, the nuclear spin sees an extra Zeeman contribution which may be tilted with respect to the z axis. Once taken into account, these corrections, the analysis, and formulas of Sect. 2.3 remain valid.

## 3 Spin systems

### 3.1 Erbium-doped ${\mathrm{CaWO}}_{4}$

The first system investigated consists of erbium ${\mathrm{Er}}^{3+}$ ions doped into a ${\mathrm{CaWO}}_{4}$ matrix, substituting ${\mathrm{Ca}}^{2+}$ . The crystal has a tetragonal body-centered structure (see Fig. [Fig Ch1.F3]) with lattice constants a=b=0.524 nm and c=1.137 nm. Rare-earth ions with an odd number of electrons such as ${\mathrm{Er}}^{3+}$ have a ground state consisting of two levels that are degenerate in zero magnetic field and separated from other levels by an energy scale equivalent to several tens of Kelvin due to the crystalline electric field and the spin–orbit interaction. This pair of electronic levels is known as a Kramers doublet and forms an effective $S_{0}=1/2$ electron-spin system, with a spin Hamiltonian $H_{\mathrm{Er}}$ [Bibr bib1.bibx2] whose form is given by Eq. ([Disp-formula Ch1.E10]).

Due to the S4 site symmetry in which rare-earth ions are found in ${\mathrm{CaWO}}_{4}$ , the g tensor is diagonal in the crystallographic frame with $g_{xx}=g_{yy}=8.38$ and $g_{zz}=1.247$ [Bibr bib1.bibx3] ( x , y , z corresponding to a , b , c ). Of all erbium atoms, 77 % are from an isotope that has nuclear spin of $I_{0}=0$ and therefore no contribution from the hyperfine term in Eq. ([Disp-formula Ch1.E10]). Their energy levels are shown in Fig. [Fig Ch1.F3] for $B_{0}$ applied in the (a,b) plane.

The remaining 23 % are from the ${}^{167}\mathrm{Er}$ isotope with $I_{0}=7/2$ . Its hyperfine coupling tensor to the ${\mathrm{Er}}^{3+}$ electron spin is diagonal, with coefficients $A_{xx}=A_{yy}=873$ MHz and $A_{zz}=130$ MHz. The 16 eigenfrequencies of the ${}^{167}\mathrm{Er}$ spin Hamiltonian are also shown in Fig. [Fig Ch1.F3], again for $B_{0}$ applied in the (a,b) plane. In the high-magnetic-field limit of $B_{0}\gg A_{\mathrm{Er}}/(g_{\mathrm{Er}}\beta_{e})$ , the eigenstates are simply described by $|\pm ,m_{I}\rangle$ , ± describing the electron-spin quantum number $m_{S}=\pm 1/2$ and $m_{I}$ the nuclear-spin quantum number. For $B_{0}<100$ mT, as is the case in the measurements described here, this limit is only approximate, but we will nevertheless use the high-field state vectors as labels for the lower-field eigenstates. The strongest EPR-allowed transitions are the $m_{I}$ -preserving transitions. In the following, we will apply the fictitious spin model with |α,β〉 = $|\pm ,m_{I}\rangle$ .

The ${\mathrm{CaWO}}_{4}$ matrix also contains nuclear spins. Indeed, the ${}^{183}W$ isotope has a spin of I=1/2 with nuclear g factor $g_{n}=0.235$ (corresponding to a gyromagnetic ratio of 1.8 MHz T ${}^{-1}$ ) and is present in a p=0.13 abundance, whereas the other tungsten isotopes are nuclear-spin free. The interaction of the ${}^{183}W$ atoms with the erbium ions gives rise to the ESEEM studied below. Because the 4f electron wave function is mainly located on the ${\mathrm{Er}}^{3+}$ ion, the contact hyperfine with the nuclear spins of the lattice is expected to be negligibly small. We therefore model the hyperfine interaction with ${}^{183}W$ by the dipole–dipole term in Eq. ([Disp-formula Ch1.E12]).

**Figure 3 Ch1.F3:**
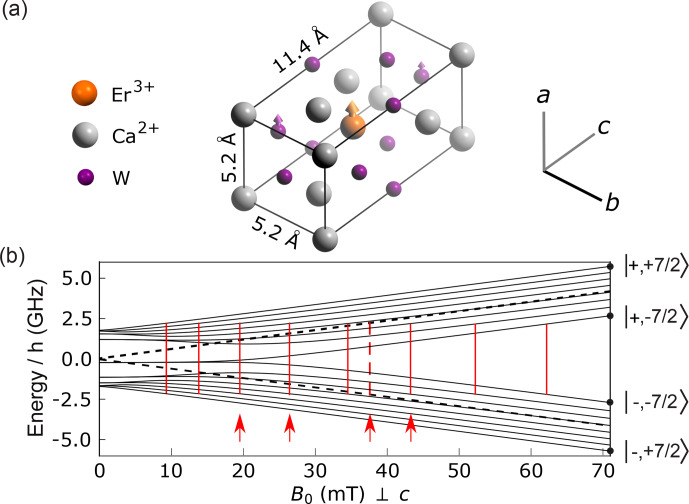
Structure and energy diagram of erbium ions in ${\mathrm{CaWO}}_{4}$ . **(a)** Crystal structure with oxygen atoms hidden for clarity. Erbium atoms are in substitution of the calcium. The crystal has a rotational symmetry around the c axis. A fraction p=0.13 of the W atoms are of the ${}^{183}W$ isotope, with a nuclear spin of 1/2 . **(b)** Energy level diagram of the I=0 erbium isotopes (dashed black line) and of the ${}^{167}\mathrm{Er}$ isotope (solid black lines) with I=7/2 for $B_{0}$ applied perpendicular to the c axis. Vertical red lines indicate the value of $B_{0}$ for which an allowed EPR transition becomes resonant with the 4.372 GHz frequency of our detection resonator (see main text, Sect. 4). Four red arrows indicate the values of $B_{0}$ at which ESEEM data were measured.

### 3.2 Bismuth donors in silicon

The other system considered is the bismuth donor in silicon. Bismuth, as an element of the fifth column, substitutes in the silicon lattice by making four covalent bonds with neighboring atoms, leaving one unpaired electron that can be weakly trapped by the hydrogenic potential generated by the ${\mathrm{Bi}}^{+}$ ion, whose spin gives rise to the resonance signal (see Fig. [Fig Ch1.F4]a). The donor wave function ψ(r) has a complex structure that extends over ≈1.5 nm in the silicon lattice [Bibr bib1.bibx21] (see the Supplement). As for $\mathrm{Er}:{\mathrm{CaWO}}_{4}$ , the donor spin Hamiltonian $H_{\mathrm{Bi}}$ is given by Eq. ([Disp-formula Ch1.E10]). However, in this case, the g tensor $g_{e}1$ is isotropic with $g_{e}=2$ , and the hyperfine tensor $A_{\mathrm{Bi}}1$ with the nuclear spin of $I_{0}=9/2$ of the bismuth atom is also isotropic, with $A_{\mathrm{Bi}}/2\pi =1.4754$ GHz.

The eigenstates of $H_{\mathrm{Bi}}$ have simple properties because of its isotropic character. Denoting $m_{S}$ ( $m_{I}$ ) the eigenvalue of $S_{z,0}$ ( $I_{z,0}$ ), we note that $m=m_{I}+m_{S}$ is a good quantum number since $H_{\mathrm{Bi}}$ commutes with $S_{z,0}+I_{z,0}$ [Bibr bib1.bibx29], z being the direction of $B_{0}$ . States with equal m are hybridized by $H_{\mathrm{Bi}}$ . States |m=5〉 and |m=-5〉 , corresponding to $|m_{S}=+1/2,m_{I}=9/2\rangle$ and $|m_{S}=-1/2,m_{I}=-9/2\rangle$ , are non-degenerate and are thus also eigenstates of $H_{\mathrm{Bi}}$ . States with |m|≤4 belong to nine two-dimensional subspaces spanned by $|m_{S}=+1/2,m_{I}=m-1/2\rangle ,|m_{S}=-1/2,m_{I}=m+1/2\rangle$ within which the two eigenstates of $H_{\mathrm{Bi}}$ are given by $|\pm ,m\rangle =a_{m}^{\pm }|\pm \frac{1}{2},m\mp \frac{1}{2}\rangle +b_{m}^{\pm }|\mp \frac{1}{2},m\pm \frac{1}{2}\rangle$ , with values of $a_{m}^{\pm },b_{m}^{\pm }$ that can be determined analytically [Bibr bib1.bibx29].

Contrary to the erbium case, the measurements of bismuth donor spins are performed in the low-field limit $\left|g_{e}\beta_{e}B_{0}\right|\ll \left|A_{\mathrm{Bi}}\right|$ , in which the eigenstates are fully hybridized. In this limit, a useful approximate expression for the eigenenergy of level |±,m〉 is

14 $$E_{m}^{\pm }\approx -\frac{A_{\mathrm{Bi}}}{2}\pm \frac{5A_{\mathrm{Bi}}}{2}\pm \frac{mg_{e}\beta_{e}|B_{0}|}{10}.$$

The magnetic-field dependence of the |±,m〉 energy levels is shown in Fig. [Fig Ch1.F4]b for $B_{0}<1$ mT. Note in particular that the separation between neighboring hyperfine levels is given by $E_{m}^{\pm }-E_{m-1}^{\pm }\approx \pm \frac{g_{e}\beta_{e}B_{0}}{10}=\pm 2\pi \times 2.8\;B_{0}$ GHz.

Because of the hybridization, all transitions that satisfy |Δm|=1 are to some extent EPR allowed at low field; i.e., they have a non-zero matrix element of operator $S_{0,x}$ . In this work, we particularly focus on the 18 |Δm|=1 transitions that are in the ≃7 GHz frequency range at low magnetic fields |+,m〉↔|-,m-1〉 and |-,m〉↔|+,m-1〉 , as shown in Fig. [Fig Ch1.F4]c. The |-,m〉↔|+,m+1〉 and |-,m+1〉↔|+,m-1〉 transitions are degenerate in frequency for -4≤m<4 as seen from Eq. ([Disp-formula Ch1.E14]), which results in only 10 different transition frequencies (see Figs. [Fig Ch1.F4]b–c and [Fig Ch1.F8]a).

The most abundant isotope of silicon is ${}^{28}\mathrm{Si}$ , which is nuclear-spin free. The lattice also contains a small percentage p of ${}^{29}\mathrm{Si}$ atoms that have a nuclear spin of I=1/2 and give rise to the ESEEM. The g factor of ${}^{29}\mathrm{Si}$ is $g_{n}=-1.11$ , yielding a gyromagnetic ratio of 8.46 MHz T ${}^{-1}$ .

The donor– ${}^{29}\mathrm{Si}$ hyperfine interaction is given by Eq. ([Disp-formula Ch1.E12]). Due to the spatial extent of the electron wave function, the Fermi contact term is not negligible and needs to be taken into account together with the dipole–dipole coupling [Bibr bib1.bibx18]; more details can be found in the Supplement.

The restriction of the total system Hamiltonian to each of the 18 ESR-allowed transitions of the bismuth donor manifold can be mapped onto the fictitious spin- 1/2 model of Sect. 2.4. Note however that the hyperfine term $\left|A_{j}\right|$ can take values up to ∼1 MHz for proximal nuclear spins, which is comparable to or larger than the frequency difference between hyperfine states of the bismuth donor manifold at low field as explained above. The validity of the fictitious spin- 1/2 model in this context will be discussed in Sect. 5.

**Figure 4 Ch1.F4:**
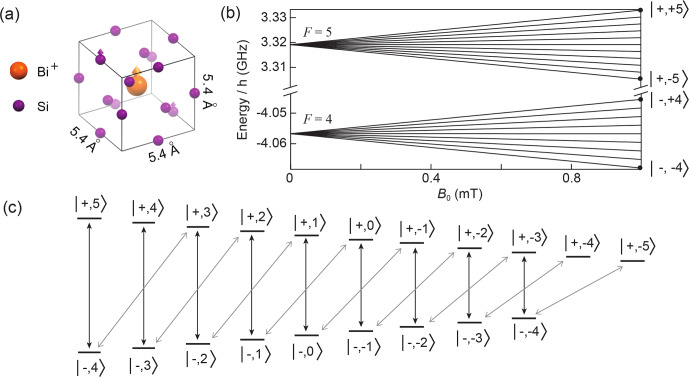
Structure and energy diagram of bismuth donors in silicon. **(a)** Silicon crystal structure, showing a substitutional bismuth atom coupled to nearby ${}^{29}\mathrm{Si}$ nuclear spins. The donor electron is trapped around the ${\mathrm{Bi}}^{+}$ ion and its wave function covers many lattice sites. **(b)** Energy levels of the bismuth donor for $B_{0}<1$ mT. **(c**) Schematic representation of the allowed transitions (black and grey arrows) between the bismuth donor energy levels in the low field limit.

## 4 Experimental setup and samples

The EPR spectrometer has been described in detail in [Bibr bib1.bibx7] and [Bibr bib1.bibx36] and is shown schematically in Fig. [Fig Ch1.F5]a. It is built around a superconducting micro-resonator of frequency $\omega_{r}$ consisting of a planar interdigitated capacitor shunted by an inductor, directly patterned on the crystal. We detect the spins that are located in the immediate vicinity of the resonator inductance. Note that the microwave $B_{1}$ field generated by the inductance is spatially inhomogeneous. If the spin location is broadly distributed, this can make the application of control pulses with a well-defined Rabi angle problematic [Bibr bib1.bibx39]. As explained below, the resonator is more strongly coupled to the measurement line than in [Bibr bib1.bibx7] to increase the measurement bandwidth as requested for ESEEM spectroscopy.

**Figure 5 Ch1.F5:**
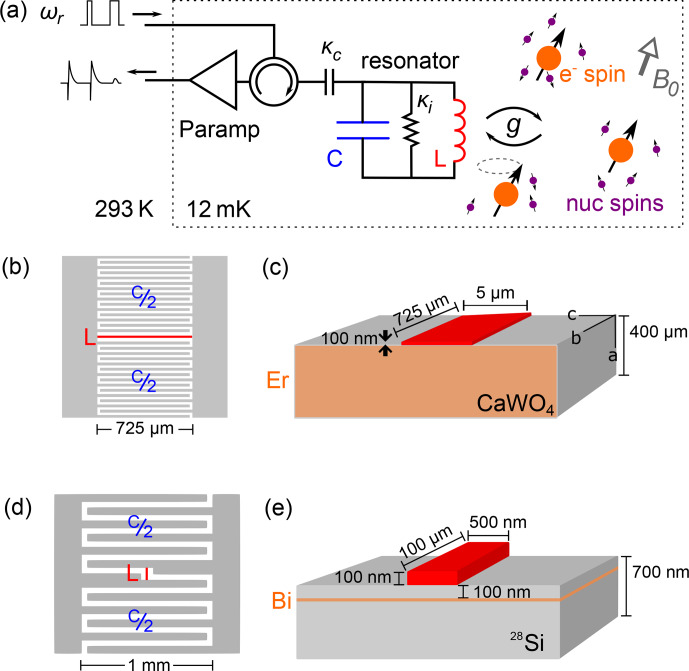
Experimental setup and samples. **(a)** Schematic of the low-temperature EPR spectrometer. The LC resonator is inductively coupled to electron spins, which are coupled to a nuclear-spin bath that causes the ESEEM. The spins are probed by sequences of microwave pulses at the resonator frequency $\omega_{r}=1/\sqrt{\text{LC}}$ . Reflected pulses as well as the echo signal are routed to a parametric amplifier, further amplified at 4 K, and finally demodulated and digitized at room temperature. **(b, c)** Design of the LC resonator used for the detection of erbium ion spins, with a 725 µm long, 5 µm wide inductor. It is patterned out of a 100 nm thick niobium film deposited on top of a ${\mathrm{CaWO}}_{4}$ substrate bulk doped with ${\mathrm{Er}}^{3+}$ ions. **(d, e)** Design of the LC resonator used for the detection of bismuth donor spins, with a 100 µm long, 0.5 µm wide inductor. It is patterned out of a 100 nm thick aluminum film deposited on top of a silicon substrate isotopically enriched in ${}^{28}\mathrm{Si}$ , in which bismuth ions were implanted at a 50–100 nm depth.

The sample is mounted in a copper sample holder thermally anchored at the mixing chamber of a dilution refrigerator. A DC magnetic field $B_{0}$ is applied parallel to the sample surface and along the resonator inductance. The resonator is coupled capacitively to an antenna, which is itself connected to a microwave measurement setup in reflection. To minimize heat load, the coaxial cables between 4 K and 10 mK are in superconducting niobium–titanium (NbTi). To suppress thermal noise, the input line is heavily attenuated at low temperatures. Microwave pulses for driving the spins are sent to the resonator input, and their reflection or transmission, together with the echo signal emitted by the spins, is fed into a superconducting JPA, either of the flux-pumped type [Bibr bib1.bibx54] or of the Josephson traveling-wave parametric amplifier (JTWPA) type [Bibr bib1.bibx23]. Further microwave amplification takes place at 4 K with a high-electron-mobility transistor (HEMT) from Low Noise Factory, and then at room temperature, before homodyne demodulation which yields the two signal quadratures [I(t),Q(t)] . The echo-containing quadrature signal is integrated to yield the echo amplitude $A_{e}$ . Such a setup was shown to reach sensitivities of the order of $10^{2}$ – $10^{3}$ spin/ $\sqrt{\mathrm{Hz}}$ [Bibr bib1.bibx7].

Because of the small resonator mode volume and high quality factor, little microwave power is needed to drive the spins. The exact amount depends on the resonator geometry, as conveniently expressed by the power-to-field conversion factor $\alpha =B_{1}/\sqrt{P_{\mathrm{in}}}$ . In the experiments reported here, the maximum microwave power used to drive the spins is on the order of 10 nW. At this power, the superconducting pre-amplifiers saturate; however, they recover rapidly enough (within a few microseconds) to amplify the much weaker subsequent spin echoes. Flux-pumped JPAs are moreover switched off during the control pulses by pulsing the pump tone, whereas the JTWPA was kept on all the time. All microwave powers reaching the 4 K HEMT are low enough that neither saturation nor damage are to be expected at this stage.

The erbium-doped sample (from Scientific Materials) was prepared by mixing erbium oxide with calcium and tungsten oxides before crystal growth, yielding a uniform Er concentration of $6\times 10^{17}$ cm ${}^{-3}$ ( 50 ppm) throughout the sample. For resonator fabrication, the bulk crystal was cut and polished to a thin rectangular sample with dimensions 0.4,mm×3mm×6,mm parallel to a×b×c axes. The resonator was patterned out of a 100 nm thick (sputtered) niobium (Nb) layer, using a design similar to that shown in [Bibr bib1.bibx7]. More specifically, 15 interdigitated fingers on either side of a 720µm×5µm inductive wire form an LC resonator, corresponding to a detection volume of $V_{\mathrm{Er}}∼20$ pL. In the absence of magnetic field, the resonance frequency is $\omega_{r}/2\pi =4.323$ GHz. Its total quality factor of $8\times 10^{3}$ is set both by the internal losses, characterized by the energy loss rate $\kappa_{i}=5\times 10^{5}$ s ${}^{-1}$ , and by its coupling to the measurement line $\kappa_{C}=3\times 10^{6}$ s ${}^{-1}$ . For this geometry, the power-to-field factor is α=1.7 T W ${}^{-1/2}$ .

The bismuth donors have been implanted at ≈100 nm depth with a peak concentration of $8\times 10^{16}$ cm ${}^{-3}$ in a silicon sample. They lie in a 700 nm thick silicon epilayer enriched in the nuclear-spin-free ${}^{28}\mathrm{Si}$ isotope (nominal concentration of 99.95 %), grown on top of a natural-abundance silicon sample. The resonator is patterned out of a 50 nm thick aluminum film. It has the same geometry as reported in [Bibr bib1.bibx36], with a 100 µm long, 500 nm wide inductor, and a detection volume of 0.2 pL. Its frequency $\omega_{r}/2\pi =7.370$ GHz is only slightly below the zero-field splitting of unperturbed Bi:Si donors at $5A_{\mathrm{Bi}}/(2\pi )=7.37585$ GHz [Bibr bib1.bibx52]. The resonator internal loss is given by $\kappa_{i}=3\times 10^{5}$ s ${}^{-1}$ . The coupling to the measurement line can be tuned at will by modifying the length of a microwave antenna that capacitively couples the measurement waveguide to the on-chip resonator via the copper sample holder [Bibr bib1.bibx7]. For the experiments reported below, we used two settings: one for which the resonator was overcoupled ( $\kappa_{C1}=10^{7}$ s ${}^{-1}$ ), corresponding to a loaded quality factor of $Q_{1}=4\times 10^{3}$ , and one for which the coupling was closer to critical ( $\kappa_{C2}=10^{6}$ s ${}^{-1}$ ), corresponding to a loaded quality factor of $Q_{2}=3.4\times 10^{4}$ . In the low- Q case, square microwave pulses were used, of duration ≃100 ns similar to the cavity field damping time. In the high- Q case, shaped pulses were used [Bibr bib1.bibx37] so that the intra-cavity field was a square pulse of 1 µs without any ringing. In some experiments, we additionally used a train of π pulses (Carr–Purcell–Meiboom–Gill sequence; CPMG), which generated extra echoes for significant gain in signal-to-noise ratio. More details on the pulse sequences used, the phase cycling scheme, and the repetition time, will be given in the following sections, together with experimental results. For this geometry, the power-to-field factor is α=9 T W ${}^{-1/2}$ for the low- Q case, and α=21 T W ${}^{-1/2}$ for the high- Q case.

## 5 Results

### 5.1 Erbium-doped ${\mathrm{CaWO}}_{4}$

#### 5.1.1 Spectroscopy

Figure [Fig Ch1.F6] shows a spectrum comprising a series of microwave transmission measurements recorded on a vector network analyzer, measured at 100 mK, as a function of the magnetic field $B_{0}$ applied along the b crystal axis [Bibr bib1.bibx38]. Note that compared to Fig. [Fig Ch1.F5]a, the resonator is coupled to the measurement line in a hanger geometry [Bibr bib1.bibx12], so that its resonance appears as a dip in the amplitude transmission coefficient $\left|S_{21}\right|$ (see Fig. [Fig Ch1.F6]). The nine red lines indicate the values of $B_{0}$ at which the calculated ${\mathrm{Er}}^{3+}$ ion transitions are equal to $\omega_{r}$ (see Fig. [Fig Ch1.F3]b). Avoided level crossings are observed, which indicate a strong coupling of the resonator to the erbium transitions. Several additional anti-crossings and discontinuities are visible above 40 mT. These are attributed to ytterbium impurities ${}^{171}\mathrm{Yb}$ and ${}^{173}\mathrm{Yb}$ and magnetic flux vortices penetrating the resonator.

Noticeable in the spectrum at 37 mT is the large anti-crossing attributed to the highly concentrated I=0 erbium isotopes. Here, the high-cooperativity regime (C>30) is reached between the electronic spins and the resonator [Bibr bib1.bibx22]. Typical linewidth of Γ/2π∼20 MHz is observed. The coupling strength is also observed to be different for the eight ${}^{167}\mathrm{Er}$ transitions, which are labeled according to their corresponding nuclear-spin projections $m_{I}$ . This is explained by the partial polarization of the ground-state hyperfine levels of ${}^{167}{\mathrm{Er}}^{3+}$ at millikelvin temperatures (see Fig. [Fig Ch1.F3]b).

**Figure 6 Ch1.F6:**
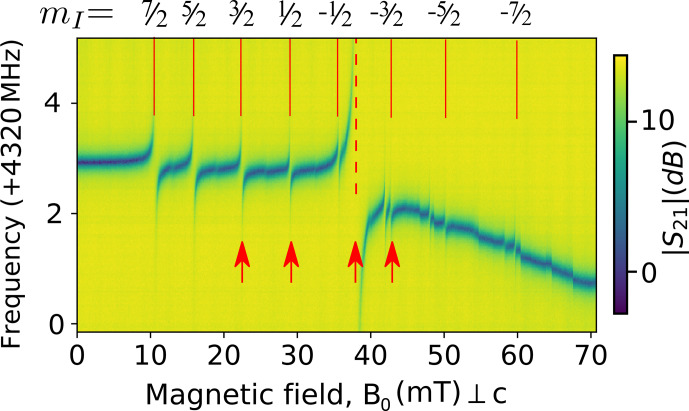
Spectroscopy of ${\mathrm{Er}}^{3+}:{\mathrm{CaWO}}_{4}$ . Transmission coefficient $\left|S_{21}|(\omega \right)$ at 100 mK as a function of the magnetic field $B_{0}$ applied along to the a crystalline axis, around 4.323 GHz. Vertical red lines indicate the expected erbium transitions either for the I=0 isotopes (dashed) or the I=7/2 isotope (solid). Red arrows indicate the field at which the ESEEM data are measured.

#### 5.1.2 Two-pulse ESEEM

Four values of $B_{0}$ were selected for investigating ESEEM, indicated by the arrows in Fig. [Fig Ch1.F6]; the first, second, and fourth corresponding to electronic-spin transitions of ${}^{167}\mathrm{Er}$ , and the third one to the I=0 isotopes. The two-pulse echo sequence of Fig. [Fig Ch1.F1]a was implemented with square pulses of 1 µs duration applied at the resonator input, with double amplitude for the second pulse. Note that due to the $B_{1}$ spatial inhomogeneity combined with the homogeneous spin distribution throughout the crystal, the spread of Rabi frequency is too large to observe a well-defined nutation signal. The Rabi angle is therefore not well defined, and the echo is the average of different rotation angles.

The control pulses driving the spins are filtered by the resonator bandwidth κ/2π≃600 kHz, corresponding to a field decay time $2\kappa^{-1}=3.3$ µs . The repetition time between echo sequences was 1 s, close to the spin relaxation time $T_{1}∼1$ –2 s measured by saturation recovery on the transitions studied. The echo signal was averaged 10 times with phase cycling of the π pulse to improve signal-to-noise ratio and to remove signal offsets.

Figure [Fig Ch1.F7] shows the two-pulse echo integrated amplitude $A_{e}$ as a function of τ for each of the four Er transitions investigated [Bibr bib1.bibx38]. A clear envelope modulation signal is observed, together with an overall damping. Here, we are interested only in the modulation pattern; a detailed study of the coherence time $T_{2}$ will be provided elsewhere. Qualitatively, we observe that the modulation frequency increases with $B_{0}$ and the modulation amplitude overall decreases with $B_{0}$ , as expected from the discussion in Sect. 2. A Fourier transform of the I=0 data (see Fig. [Fig Ch1.F7]b) shows the ESEEM spectrum. Well-resolved peaks are observed in the 5–100 kHz range, distributed around the ${}^{183}W$ bare Larmor frequency $\omega_{W}$ .

A very rough estimate of the number of erbium ions contributing to the signal is $[\mathrm{Er}]V_{\mathrm{Er}}\kappa /\Gamma$ , which is $2.5\times 10^{8}$ for the I=0 data, and $10^{7}$ for each ${}^{167}\mathrm{Er}$ transition.

**Figure 7 Ch1.F7:**
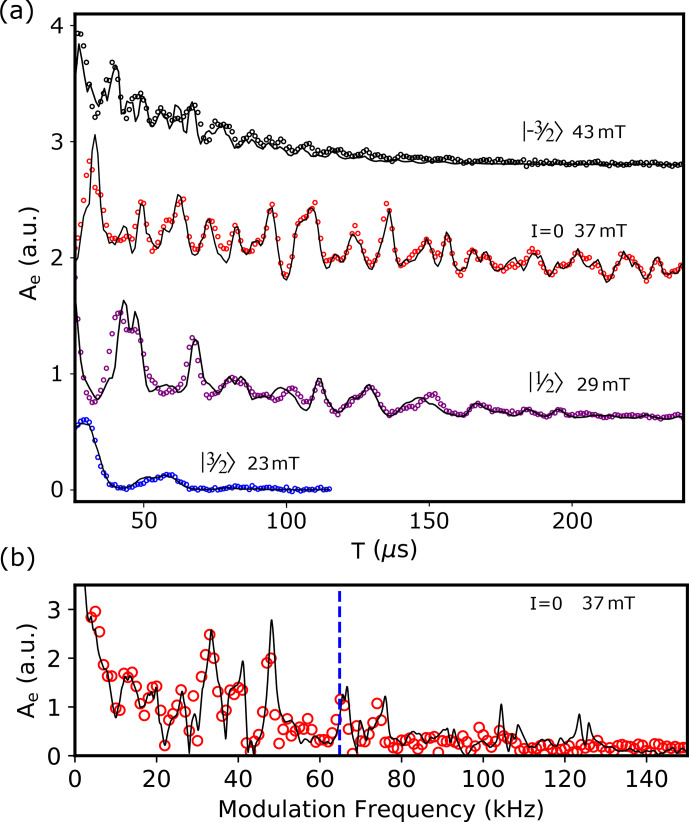
Two-pulse ESEEM on $\mathrm{Er}:{\mathrm{CaWO}}_{4}$ . **(a)** Integrated echo area $A_{e}$ as a function of the inter-pulse delay τ for four values of $B_{0}$ corresponding to different transitions. Open circles are measurements, and solid lines are the results of the ESEEM calculations as explained in Sect. S5.1. **(b)** Measured (open red circles) and computed (solid line) fast Fourier transform of the I=0 data. The dashed blue line shows the Larmor frequency of ${}^{183}W$ nuclei in free space.

#### 5.1.3 Comparison with the model

We compute the echo envelope $V_{2p}^{\prime }(\tau )$ described in Sect. 2.3, with the nearest 1000 coupled tungsten nuclei (N=1000) and a natural ${}^{183}W$ abundance of 14.4 % (p=0.144) . The hyperfine interaction is taken to be purely dipolar, as already explained [Bibr bib1.bibx17]. The fitting proceeds by assigning an initial “guess” to six free parameters, then minimizing using the limited-memory Broyden–Fletcher–Goldfarb–Shanno algorithm for bound-constrained optimization (L-BFGS-B) [Bibr bib1.bibx9]. Three of these parameters $\left(|B_{0}|,\phi ,\theta \right)$ describe the applied magnetic field:

$$B_{0}=|B_{0}|\left[\mathrm{sin}\theta \mathrm{cos}\phi \;x+\mathrm{sin}\theta \mathrm{sin}\phi \;y+\mathrm{cos}\theta \;z\right].$$

Here, θ is the angle of the field relative to the crystal c axis (z) and ϕ is the angle relative to the a axis (x) in the a – b plane ( x – y plane). The other three parameters ( C , $T_{2}$ , n ) account for the echo envelope decay:

$$1A_{e}(\tau )=V_{2p}(\tau )\cdot C\mathrm{exp}{\left(-\frac{2\tau }{T_{2}}\right)}^{n},$$

where C represents the signal magnitude, $T_{2}$ represents the coherence time, and n∈[1,2] accounts for non-exponential decay. To determine the global minimum of the fit, the minimization is repeated 200 times with randomly seeded initial values for the six parameters, bounded within the known uncertainty of the applied magnetic field $B_{0}$ , signal strength C , and coherence time $T_{2}$ . This approach reveals single local minima for each fitted parameter within the bounded range, with the variance of the 200 outcomes determining the uncertainty for each parameter. In particular, it yields precise values for the angles θ=91.47±0.01 ${}^{\circ }$ and ϕ=90.50±0.01 ${}^{\circ }$ . The result of this fitting is presented in Fig. [Fig Ch1.F7]a, overlaid on the data for the I=0 transition at 37 mT. Only the decay parameters ( C , $T_{2}$ , n ) and magnetic-field magnitude $\left|B_{0}\right|$ are left free when fitting the other three transitions in Fig. [Fig Ch1.F7]a. This was done for consistency between datasets, and because the I=0 data yield the most accurate values for ϕ and θ due to the low decoherence rate. The fits yield coherence times $T_{2}$ varying between 40 and 400 µs , depending on the transition considered. Good agreement was also reached between the fitted and expected (pre-calibrated) field magnitudes.

Note that good fits to the data are also achieved by including only the nearest 100 tungsten nuclei, although noticeable deviations between the data and fit are observed with any amount less. The dimensionless “anisotropic hyperfine interaction parameter” ρ described in the seminal publication on ESEEM [Bibr bib1.bibx41] is not required here. This parameter was introduced with the earliest attempts of ESEEM fitting, likely to compensate for the low number of simulated nuclear spins (typically 10 nearest nuclei or less) and was interpreted as an account for a potential distortion of the local environment caused by dopant insertion. Finally, a consideration of the spectral components presented in Fig. [Fig Ch1.F7]b helps to more clearly identify the difference between the fit and the data. In particular, the high-frequency components of the fitted model are not present experimentally due to the filtering effect of the superconducting resonance (260 kHz half width at half maximum; HWHM). This high- Q resonator greatly reduces the bandwidth of the radio frequency (RF) field absorbed by the coupled $\mathrm{Er}{-}^{183}W$ system and further limits the bandwidth of the detected echo signal.

### 5.2 Bismuth donor sample

#### 5.2.1 Spectroscopy

Given the resonator frequency $\omega_{r}$ , four bismuth donor resonances should be observed when varying $B_{0}$ between 0 and 1 mT, as seen in Fig. [Fig Ch1.F8]a. Figure [Fig Ch1.F8]b shows an echo-detected field sweep, measured at 12 mK: the integrated area $A_{e}$ of echoes obtained with a sequence shown in Fig. [Fig Ch1.F1]a with τ=50 µs pulse separation is plotted as a function of $B_{0}$ [Bibr bib1.bibx38]. Instead of showing well-separated peaks as in the erbium case, echoes are observed for all fields below 1 mT, with a maximum close to 0.1 mT, and extends in particular down to $B_{0}=0$ mT . This is the sign that each of the expected peaks is broadened and overlaps with neighboring transitions. Close to zero field, the echo amplitude goes down by a factor of 2 on a scale of ∼0.1 mT, before showing a sharp increase at exactly zero field. These zero-field features are not currently understood, but they are reproducible as confirmed by the measurements at $B_{0}<0$ , which are approximately symmetric to the $B_{0}>0$ data as they should be.

Line broadening was reported previously for bismuth donors in silicon in related experiments [Bibr bib1.bibx7] and was attributed to the mechanical strain exerted by the aluminum resonator onto the silicon substrate due to differential thermal contractions between the metal and the substrate. At low strain, $A_{\mathrm{Bi}}$ depends linearly on the hydrostatic component of the strain tensor $\epsilon_{\mathrm{hs}}=(\epsilon_{xx}+\epsilon_{yy}+\epsilon_{zz})/3$ with a coefficient $dA_{\mathrm{Bi}}/d\epsilon_{\mathrm{hs}}/(2\pi )=28$ GHz [Bibr bib1.bibx25]. Quantitative understanding of the line shape was achieved in a given sample geometry based on this mechanism [Bibr bib1.bibx32], using a finite-element modeling to estimate the strain profile induced upon sample cooldown. A similar modeling was performed for the Bi sample reported here (see Fig. [Fig Ch1.F8]d). Based on the typical strain distribution $|\epsilon_{\mathrm{hyd}}|∼3\times 10^{-4}$ and on the hyperfine to strain coefficient $dA_{\mathrm{Bi}}/d\epsilon_{\mathrm{hs}}/(2\pi )=28$ GHz, we expect the zero-field splitting $5A_{\mathrm{Bi}}/(2\pi )$ to have a spread of ∼50 MHz, which would indeed result in complete peak overlap in the $B_{0}<1$ mT region, as observed in Fig. [Fig Ch1.F8]b.

This broadening has two consequences worth highlighting. First, the bismuth donor echo signals can be measured down to $B_{0}=0$ mT, which otherwise is generally impossible in X-band spectroscopy. Here, this is enabled by the large hyperfine coupling of the Bi:Si donor, combined with strain-induced broadening. This makes it possible to detect ESEEM caused by very weakly coupled nuclear spins, which requires low magnetic fields as explained in Sect. 2. Second, at a given magnetic field, the spin-echo signal contains contributions from several overlapping EPR transitions. This last point is best understood from Fig. [Fig Ch1.F8]c, which shows how several classes of bismuth donors, each with different hyperfine coupling $A_{\mathrm{Bi}}$ , may have transitions resonant with $\omega_{r}$ . We will assume in the following that the inhomogeneous distribution of $A_{\mathrm{Bi}}$ is so broad that each of the 10 $A_{\mathrm{Bi}}$ values for which one bismuth donor transition is resonant with $\omega_{r}$ at fixed $B_{0}$ is equally probable, which is likely to be valid for $B_{0}<1$ mT.

**Figure 8 Ch1.F8:**
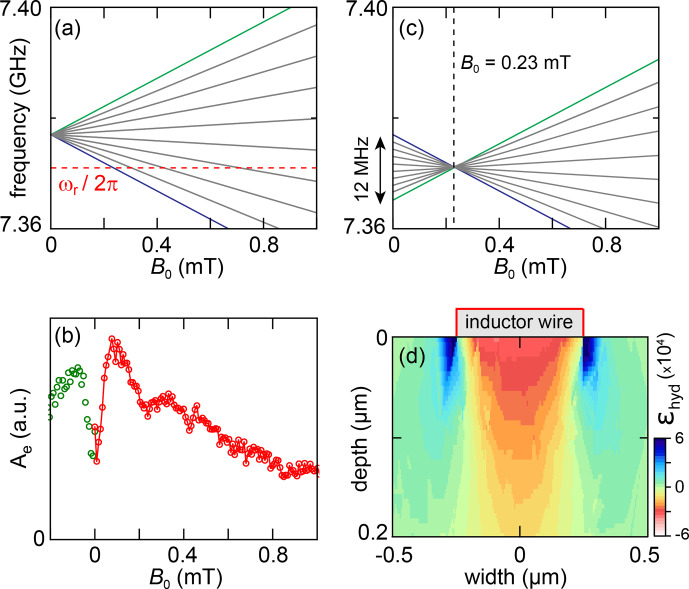
**(a)** EPR-allowed transitions of a bismuth donor in silicon for $0<B_{0}<1$ mT. The dashed red line denotes the resonator frequency $\omega_{r}$ . The spectrum is for an unstrained donor for which the frequency at $B_{0}=0$ is $5A_{\mathrm{Bi}}/(2\pi )$ . **(b)** Echo-detected field sweep. The echo integral $A_{e}$ is plotted versus $B_{0}$ . **(c)** Frequency of all 18 bismuth donor transitions that may contribute to the echo signal at a given field (here, $B_{0}=0.23$ mT). This is made possible by the strain-induced spread in $A_{\mathrm{Bi}}$ between different donors. **(d)** Hydrostatic component of strain in silicon simulated using COMSOL.

#### 5.2.2 Two-pulse ESEEM

Two-pulse echoes are measured with the pulse sequence shown in Fig. [Fig Ch1.F1], which consists of a square $\pi /2_{X}$ pulse of duration 50 ns followed by a square $\pi_{Y}$ pulse of duration 100 ns after a delay τ . Note that due to the donor spatial location in a shallow layer below the surface and to the strain shifting of their Larmor frequency [Bibr bib1.bibx32], the Rabi frequency is more homogeneous than in the erbium-doped sample, and Rabi rotations with a well-defined angle can be applied [Bibr bib1.bibx32]. To increase the signal-to-noise ratio, a CPMG sequence of 198 π pulses separated by 10 µs are used following the echo sequence [Bibr bib1.bibx36]. The curves are repeated 20 times, with a delay of 2 s in between to enable spin relaxation of the donors. All the resulting echoes are then averaged. Phase cycling is performed by alternating sequences with opposite phases for the π/2 pulses and subtracting the resulting echoes. The data are obtained in the low- Q configuration (see Sect. 4).

**Figure 9 Ch1.F9:**
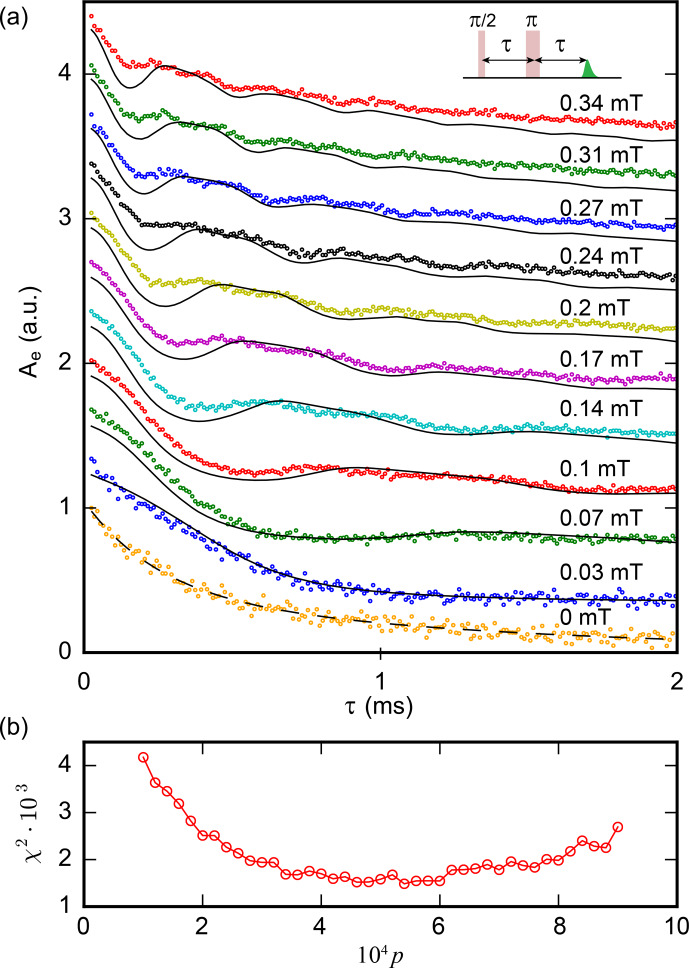
Two-pulse ESEEM of Bi:Si donors. **(a)** Echo integral $A_{e}$ versus inter-pulse delay τ for a two-pulse echo sequence for varying magnetic field $B_{0}$ . Dots indicate experimental data; lines indicate results of the model (see text), assuming a concentration in ${}^{29}\mathrm{Si}$ of $p=4.4\times 10^{-4}$ . The curves are vertically shifted for clarity. **(b)** Fit residue $\chi^{2}$ for different ${}^{29}\mathrm{Si}$ relative abundance p . The best fit is obtained for $p=4.4\pm 1\times 10^{-4}$ , in agreement with the specified value.

Figure [Fig Ch1.F9] shows the integral of the averaged echoes $A_{e}(\tau )$ as a function of τ for various values of $B_{0}$ [Bibr bib1.bibx38]. At a non-zero field, $A_{e}(\tau )$ shows $B_{0}$ -dependent oscillations on top of an exponential decay with time constant $T_{2}=2.6$ ms. Similar decay times were measured on the same chip with another resonator [Bibr bib1.bibx36], and are attributed to a combination of donor–donor dipolar interactions and magnetic noise from defects at the sample surface.

**Figure 10 Ch1.F10:**
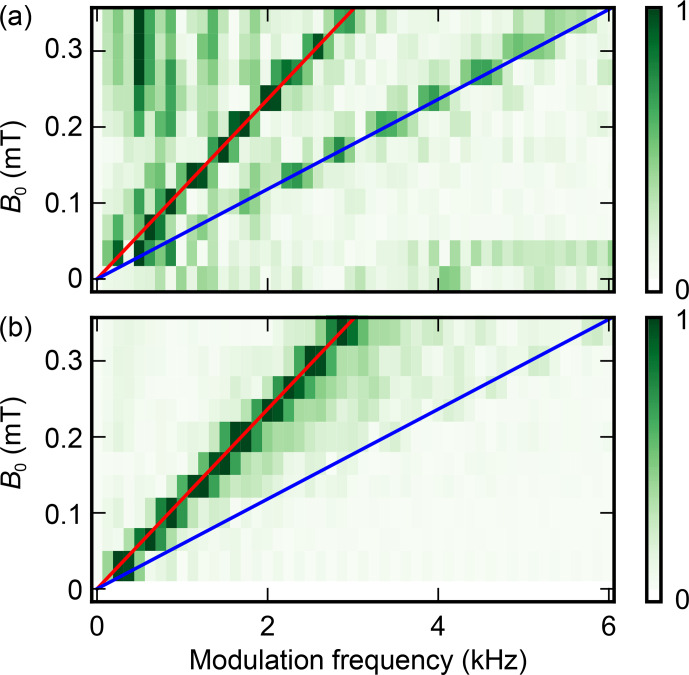
Amplitude of the Fourier transform of the experimental **(a)** and theoretical **(b)** two-pulse Bi:Si donor ESEEM data.

In the subsequent discussion, we concentrate on the ESEEM pattern. To analyze the data, each curve was divided by a constant exponential decay with 2.6 ms time constant, mirrored at t=0 , and Fourier transformed (see Fig. [Fig Ch1.F10]). Only two peaks are observed. Their frequencies vary linearly with $B_{0}$ , and are found to be approximately 8 and 16 kHz mT ${}^{-1}$ . This is in good agreement with the gyromagnetic ratio of ${}^{29}\mathrm{Si}$ ( 8.46 kHz mT ${}^{-1}$ ); the presence of the second peak at twice this value is expected as explained in Sect. 2 for the two-pulse ESEEM in the weak-coupling limit. The oscillation amplitude goes down with $B_{0}$ , again as expected from the model put forward in Sect. 2.

A rough estimate of the number of donors contributing to the measurements shown in Fig. [Fig Ch1.F9] can be obtained by comparison with [Bibr bib1.bibx36]. Given the nearly identical resonator geometry, and assuming identical strain broadening in both samples, the ratio of the number of donors involved in both measurements is simply given by the ratio of resonator bandwidths. For the low- Q configuration, such as the two-pulse echo of Fig. [Fig Ch1.F9], this corresponds to $≃5\times 10^{3}$ dopants; in the high- Q configuration (see the three- and five-pulse data in the next paragraph), this number is reduced to $≃5\times 10^{2}$ dopants.

#### 5.2.3 Three- and five-pulse ESEEM

The spectral resolution provided by the measurement protocol is limited because of the finite electron coherence time $T_{2}$ . As discussed in Sect. 2.3, this can be overcome by three- or five-pulse ESEEM.

**Figure 11 Ch1.F11:**
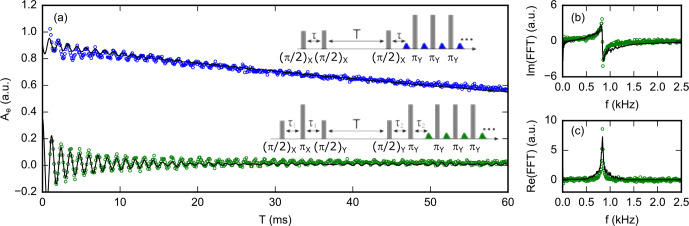
**(a)** Three-pulse (blue circles) and five-pulse (green circles) ESEEM signals of Bi:Si donors at $B_{0}=0.1$ mT. Black lines are simulations assuming a ${}^{29}\mathrm{Si}$ concentration of $p=4.4\times 10^{-4}$ . **(b)** Imaginary and **(c)** real part of the Fourier transform of the five-pulse ESEEM data. The spectrum only contains a peak at 850 Hz, which is the ${}^{29}\mathrm{Si}$ nuclei Larmor frequency at this field.

We measure three- and five-pulse ESEEM with the pulse sequence shown in Fig. [Fig Ch1.F11]. The high- Q configuration is chosen for which $T_{1}=120$ ms is measured (see the Supplement); shaped pulses generate an intra-cavity field in the form of a rectangular pulse of 1 µs duration with sharp rise and fall [Bibr bib1.bibx37] despite the high resonator quality factor. The data are acquired at $B_{0}=0.1$ mT, so that $\omega_{I}/2\pi ≃850$ Hz. The first blind spot for three-pulse ESEEM is thus at $2\pi /\omega_{I}=1.2$ ms; we chose τ=290 µs for the three-pulse echo, and $\tau_{1}=\tau_{2}=290$ µs for the five-pulse sequence. A sequence of 19 CPMG π pulses, separated by 50 µs , was used to enhance the signal-to-noise ratio. The sequences were repeated after a fixed waiting time of 100 ms between the last π pulse of one sequence and the first π/2 pulse of the following, to enable spin relaxation. Phase cycling is used to suppress unwanted echoes (see the Supplement for the schemes; [Bibr bib1.bibx44]). Each point is averaged over $2.5\times 10^{4}$ sequences, with a total acquisition time of 2 weeks for each curve [Bibr bib1.bibx38].

The results are shown in Fig. [Fig Ch1.F11], together with their fast Fourier transform [Bibr bib1.bibx38]. Both the three-pulse ESEEM (3PE) and five-pulse ESEEM (5PE) curves show oscillations that last 1 order of magnitude longer than the electron spin $T_{2}$ (up to 20 ms), enabling higher spectral resolution of the ESEEM signal. The 5PE curve has a higher oscillation amplitude than the 3PE by a factor of 2–3, as expected. The decay of the oscillations occurs in ∼10 ms, 1 order of magnitude faster than the stimulated echo amplitude (see the 3 PE curve), suggesting that it is an intrinsic feature of the ESEEM signal, as discussed below.

The spectrum shows only one peak at the ${}^{29}\mathrm{Si}$ frequency. This is consistent with the expression provided in Sect. 2 and the Supplement for the three- and five-pulse ESEEM, in which the terms oscillating at the sum and difference frequency are absent in contrast to the two-pulse ESEEM. The peak width is ≃100 Hz, which indicates that the nuclei contributing to the ESEEM signal have hyperfine coupling strengths A , B of at most 100 Hz. Neglecting the contact interaction term, this corresponds to ${}^{29}\mathrm{Si}$ nuclei that are located at least ∼5 nm away from the donor spin.

The measured ESEEM spectrum of the bismuth donor sample qualitatively differs from the erbium sample, since it only contains a peak at the unperturbed silicon nuclei Larmor frequency (and at twice this frequency for the two-pulse ESEEM), instead of the many peaks observed in Fig. [Fig Ch1.F7] indicating nuclear-spin contribution with vastly different hyperfine strengths. This can be qualitatively understood by examining Eq. ([Disp-formula Ch1.E8]). Defining $N_{l}$ as the number of lattice sites with approximately the same hyperfine parameters $A_{l}$ , $B_{l}$ and modulation frequency $\omega_{↓/↑,l}$ , the component at $\omega_{↓/↑,l}$ is visible in the spectrum if $N_{l}k_{l}p∼1$ , which can only be achieved if $N_{l}p∼1$ . In the case of erbium, p=0.144 so that even the sites closest to the ion (for which $N_{l}$ is of order unity) may satisfy this condition for well-chosen $B_{0}$ . In the bismuth donor sample where $p=4.4\times 10^{-4}$ , this condition can only be met for $N_{l}∼10^{3}$ , and therefore for crystal sites l that are far from the donor, for which the hyperfine coupling is small, so that $\omega_{↓/↑,l}≃\omega_{I}$ . This is confirmed by the more quantitative modeling below.

#### 5.2.4 Comparison with the model

As explained above, the measured echo signal results from the contribution of all 18 Bi:Si transitions because of strain broadening. To model the data, we therefore apply the fictitious spin- 1/2 model to each transition, and sum the resulting echo amplitudes weighted by their relative contribution, which we determine using numerical simulations described in the Supplement.

Moreover, as discussed in Sect. 3.2, and in contrast to the erbium case, the fictitious spin model for a given transition needs to be validated in the low- $B_{0}$ regime because the energy difference between neighboring hyperfine levels of the bismuth donor manifold ( $E_{m}^{\pm }-E_{m-1}^{\pm })/h≃0.3$ MHz for $B_{0}=0.1$ mT is comparable to or even lower than the hyperfine coupling to some ${}^{29}\mathrm{Si}$ nuclei. In that case, the hyperfine interaction induces significant mixing between the bismuth donor and the ${}^{29}\mathrm{Si}$ eigenstates, and we should describe the coupled “electron spin $S_{0}+{}^{209}\mathrm{Bi}$ nuclear spin $I_{0}+{}^{29}\mathrm{Si}$ nuclear spin I” system as a single 40 -level quantum system.

This study is described in Sect. S5 for a ${}^{29}\mathrm{Si}$ with strong hyperfine coupling ( ≥200 kHz). The state mixing makes many transitions EPR allowed, and the interference between these transitions causes fast oscillations in the spin-echo signal, as seen in Fig. S7 in the Supplement. The frequencies of these oscillations depend greatly on the local Overhauser field on the donor electron spin. Since the latter has a large inhomogeneous broadening ( ∼0.5 MHz), the ensemble average leads to a rapid decay of the signal ( <1 µs ). Given the ${}^{29}\mathrm{Si}$ concentration, about 10 % of the donors have one or more ${}^{29}\mathrm{Si}$ with coupling >300 kHz in the proximity, which therefore leads to a rapid decay of the total echo signal within ∼1 µs by about 10 %. In the experimental data, this fast decay is not visible because the echo signal is measured at longer times, and therefore the ESEEM signals presented in Fig. S5 are those from ${}^{29}\mathrm{Si}$ with couplings <200 kHz.

As for spins with a coupling strength between 20 and 200 kHz, they lead to ESEEM amplitude much less than 1 %, as shown in Figs. S7–S9. For nuclear spins with a hyperfine coupling <100 kHz, the fictitious spin model produces results with negligible errors of the modulation frequencies from the exact solution (Figs. S5 and S6). Furthermore, the systematic numerical studies (Figs. S9) show that a nearby Si nuclear spin with coupling <100 kHz has little effect on the ESEEM due to other distant nuclear spins.

Considering these different contributions of Si nuclear spins of different hyperfine couplings, as discussed in the paragraph above and in more details in the Supplement, we apply the fictitious spin- 1/2 model to each EPR-allowed transition of the bismuth donor manifold, considering only Si nuclear spins that have a hyperfine coupling weaker than a certain cut-off which we choose as 20 kHz, and discarding all the others.

For each transition, we compute the hyperfine parameters that enter the fictitious spin- 1/2 model for all sites of the silicon lattice. We then generate a large number of random configurations of nuclear spins. We compute the corresponding two-, three-, or five-pulse ESEEM signal using the analytical formulas of Sect. 2.4 after discarding all nuclei whose hyperfine coupling is larger than 20 kHz. We average the signal for one configuration over all bismuth donor transitions using the weights determined by simulation and then average the results over all the configurations computed. In this way, we obtain the curves shown in Fig. [Fig Ch1.F9].

We use the two-pulse echo dataset to determine the most likely sample concentration in ${}^{29}\mathrm{Si}$ , using p as a fitting parameter. As seen in Fig. [Fig Ch1.F9]b, the best fit is obtained for $p=4.4\pm 1\times 10^{-4}$ , which is compatible with the specified $5\times 10^{-4}$ . The agreement is satisfactory but not perfect, as seen, for instance, in the amplitude of the short-time ESEEM oscillations which are lower in the measurements than in the simulations, particularly at larger field. Also, the peak at $2\omega_{I}$ is notably broader and has a lower amplitude than in the experiment.

For the fitted value of p , the three- and five-pulse theoretical signals are also computed and found to be in overall agreement with the data, even though the decay of the ESEEM signal predicted by the model is faster than in the experiment and correspondingly the predicted ESEEM spectrum broader than the data.

## 6 Discussion and conclusion

We have reported two-, three-, and five-pulse ESEEM measurements using a quantum-limited EPR spectrometer on two model systems: erbium ions in a ${\mathrm{CaWO}}_{4}$ matrix and bismuth donors in silicon. Whereas the erbium measurements are done in a commonly used regime of high field, the bismuth donor measurements are performed in an unusual regime of low nuclear-spin density, low hyperfine coupling, and almost zero magnetic field. Good agreement is found with the simplest analytical ESEEM models.

Having demonstrated that ESEEM is feasible in a millikelvin quantum-limited EPR spectrometer setup on two model spin systems, it is worth speculating in broader terms about its potential for real-world hyperfine spectroscopy. First, high magnetic fields are desirable for a better spectral resolution. Superconducting resonators in Nb, niobium nitride (NbN), or niobium titanium nitride (NbTiN) can retain a high quality factor up to ∼1 T [Bibr bib1.bibx16], so that quantum-limited EPR spectroscopy in the Q band can in principle be envisioned. Resonator bandwidths larger than demonstrated here are also desirable. Given, increasing κ in the Purcell regime leads to longer relaxation times $T_{1}$ , this should be done with care. One option is to increase also the coupling constant g by further reduction of the resonator mode volume [Bibr bib1.bibx40]. Interestingly, this provides another motivation to apply higher magnetic fields, since g is proportional to $\omega_{r}$ . Overall, a resonator at $\omega_{r}/2\pi ≃30$ GHz, in a magnetic field $B_{0}≃1$ T, and with a κ/2π∼10 MHz bandwidth seems within reach, while keeping the Purcell $T_{1}$ well below 1 s. Such a high-bandwidth, high-sensitivity EPR spectrometer would be ideally suited for studying surface defects. One potential concern, however, is the power-handling capability of the resonator, as the kinetic inductance causes a non-linear response at high power.

## Supplement

10.5194/mr-1-315-2020-supplement The supplement related to this article is available online at: https://doi.org/10.5194/mr-1-315-2020-supplement.

## Data Availability

All code and data necessary for generating Figs. 6–11 can be found at https://doi.org/10.7910/DVN/ZJ2EEX (Probst et al., 2020). The analysis and plotting code is written in Python (.py) and Igor (.pxp). These files are sorted according to figure number, with the relevant files for each figure compressed into a single 7zip file (.7z).
