# Supplementary material for: Hyperfine spectroscopy in a quantum-limited spectrometer
Source: Magn Reson (Gott). 2020 Dec 17;1(2):315–30. doi: 10.5194/mr-1-315-2020 (PMC10500700; doi:10.5194/mr-1-315-2020)
Supplement: The supplement related to this article is available online at: https://doi.org/10.5194/mr-1-315-2020-supplement. [file mr-1-315-supplement.pdf]

Supplement of Magn. Reson., 1, 315–330, 2020
https://doi.org/10.5194/mr-1-315-2020-supplement
© Author(s) 2020. This work is distributed under
the Creative Commons Attribution 4.0 License.

Open Access
MAGNETIC
RESONANCE

Supplement of

Hyperfine spectroscopy in a quantum-limited spectrometer

Sebastian Probst et al.

Correspondence to: Patrice Bertet (patrice.bertet@cea.fr)

The copyright of individual parts of the supplement might differ from the CC BY 4.0 License.

Section S1 - Details of the Model for ESEEM of Bi:Si

In this Section, we give the details of the Si:Bi Hamiltonian, as a supplementary note to Section III B of the main text.

The Hamiltonian of the bismuth donor spins and the ^{29}Si nuclear spins is

$$H = H_{\rm Bi} + H_{\rm Si} + H_{\rm hf}, \tag{S1}$$

where $H_{\rm Bi}$ is the Hamiltonian for the Bismuth electron and nuclear spin, $H_{\rm Si}$ is the Zeeman energy and the dipole-dipole interaction of the ^{29}Si nuclear spins, and $H_{\rm hf}$ is the hyperfine interaction between the Bismuth electron spin and the ^{29}Si nuclear spins.

The Hamiltonian of the Bi center spins is (Ma et al., 2015)

$$H_{\rm Bi} = g_{\rm e}\beta_{\rm e} S_{0,z} + A_{\rm Bi}\mathbf{S}_0 \cdot \mathbf{I}_0, \tag{S2}$$

where the notations are the same as in main text. The electron gyromagnetic ratio $g_{\rm e}\beta_{\rm e} = 1.76\times 10^{11}\,{\rm S}^{-1}{\rm T}^{-1}$ and the Fermi contact hyperfine coupling $A_{\rm Bi}/2\pi = 1.4754\,{\rm GHz}$. Note that the Zeeman energy of the nuclear spin is neglected (as explained in the main text).

The internal Hamiltonian of the ^{29}Si nuclear spin bath is

$$H_{\rm Si} = \omega_I \sum_j I_{j,z} + \sum_{i<j} \mathbf{I}_i \cdot \frac{\mu_0 g_{\rm n}^2 \beta_n^2}{4\pi r_{ij}^3}\left(1 - \frac{3\mathbf{r}_{ij}\mathbf{r}_{ij}}{r_{ij}^2}\right)\cdot \mathbf{I}_j,$$

where $\omega_I = g_{\rm n}\beta_n B_0$ denotes the Larmor frequency of the ^{29}Si nuclear spin with the gyromagnetic ratio $g_{\rm n}\beta_n = -5.319\times 10^7{\rm s}^{-1}{\rm T}^{-1}$, the spin operator $\mathbf{I}_j$ denotes the nuclear spin at position $\mathbf{r}_j$, and $\mathbf{r}_{ij} = \mathbf{r}_i - \mathbf{r}_j$.

The hyperfine interaction between the electron spin and the ^{29}Si nuclear spins is

$$H_{\rm hf} = \sum_j \mathbf{S}_0 \cdot \bar{\mathbf{A}}_j \cdot \mathbf{I}_j \equiv \sum_j H_{{\rm hf},j}, \tag{S3}$$

where the hyperfine coupling tensor includes both the Fermi contact term $A_{{\rm cf},j}$ (a scalar) and the dipolar interaction $\bar{\mathbf{A}}_{{\rm dd},j}$, i.e., $\bar{\mathbf{A}}_j = A_{{\rm cf},j}\mathbf{1} + \bar{\mathbf{A}}_{{\rm dd},j}$. The dipolar hyperfine coupling tensor is

$$\bar{\mathbf{A}}_{{\rm dd},j} = \frac{\mu_0 g_{\rm e}\beta_{\rm e} g_{\rm n}\beta_{\rm n}}{4\pi r_j^3}\left(1 - \frac{3\mathbf{r}_j\mathbf{r}_j}{r_j^2}\right) \equiv A_{{\rm dd},j}\left(1 - \frac{3\mathbf{r}_j\mathbf{r}_j}{r_j^2}\right),$$

decaying with a cubic power of the distance. The Fermi contact interaction is proportional to the electron density $|\psi(\mathbf{r}_j)|^2$ of the Bismuth donor at the nuclear spin position $\mathbf{r}_j$ (Feher, 1959; Kohn, 1957; de Sousa and Sarma, 2003),

$$A_{{\rm cf},j} = \frac{2}{3}\mu_0 g_{\rm e}\beta_{\rm e} g_{\rm n}\beta_{\rm n}|\psi(\mathbf{r}_j)|^2.$$

The electron density

$$|\psi(\mathbf{r}_j)|^2 = \frac{2}{3}\eta |f(\mathbf{r}_j)|^2 \left[\cos(k_0 x_j) + \cos(k_0 y_j) + \cos(k_0 z_j)\right]^2,$$

where $k_0 = 0.85\frac{2\pi}{a_{\rm Si}}$ (with $a_{\rm Si} = 0.543\,{\rm nm}$) is the wavenumber of the conduction band minimum, $\eta \approx 180$ is the charge density on each site, and the envelope function is taken as the Kohn-Luttinger wave function form (de Sousa and Sarma, 2003)

$$f_j(\mathbf{r}) = \frac{1}{\sqrt{\pi (sa)^2 (sb)}} \exp\left(-\sqrt{\frac{z^2}{(sb)^2} + \frac{x^2+y^2}{(sa)^2}}\right),$$

with $a = 2.51\,{\rm nm}$ and $b = 1.44\,{\rm nm}$ being the characteristic lengths for hydrogenic impurities in Si and the scaling factor $s = 0.64$ for bismuth (Hale and Mieher, 1969). The hyperfine coupling strength $|\bar{\mathbf{A}}_j|$ is mostly $<<$ MHz.

Section S2 - Applying the fictitious spin-1/2 model to Bi:Si coupled to ^{29}Si spins

In this section, we provide detailed justifications for applying the fictitious model in Section II D of main text to Bi:Si coupled to ^{29}Si spins.

The eigenstates $|\pm,m\rangle$ of the Bismuth Hamiltonian $H_{\rm Bi}$ in Eq. (S2) are

$$|\pm,m\rangle = \cos\frac{\theta_m}{2}|\pm\frac{1}{2},m\mp\frac{1}{2}\rangle \pm \sin\frac{\theta_m}{2}|\mp\frac{1}{2},m\pm\frac{1}{2}\rangle, \tag{S4}$$

with $\tan\theta_m = \frac{\sqrt{25-m^2}}{m+(1+\delta)g_{\rm e}\beta_{\rm e}B_0/A_{\rm Bi}}$, and the corresponding eigenenergies are

$$E_m^{\pm} = -\frac{A_{\rm Bi}}{4} \pm \frac{A_{\rm Bi}}{2}\sqrt{\left(m+\frac{g_{\rm e}\beta_{\rm e}B_0}{A_{\rm Bi}}\right)^2 + 25 - m^2}. \tag{S5}$$

For the experimental condition $|g_{\rm e}\beta_{\rm e}B_0| \ll |A_{\rm Bi}|$, the eigenenergies in Eq. (S5) can be approximated as

$$E_m^{\pm} \approx -\frac{A_{\rm Bi}}{2} \pm \frac{5A_{\rm Bi}}{2} \pm \frac{m g_{\rm e}\beta_{\rm e}B_0}{10},$$

and the level splitting

$$E_m^{\pm} - E_{m-1}^{\pm} \approx \pm\frac{g_{\rm e}\beta_{\rm e}B_0}{10}.$$

For a field about 1 Gauss (a typical value in our experiments), $(2\pi)^{-1}g_{\rm e}\beta_{\rm e}B_0/10 \sim 300$ kHz.

The hyperfine coupling to the ^{29}Si nuclear spins can be written in the basis of the eigenstates of $H_{\rm Bi}$. The non-vanishing matrix elements of the electron spin operators are

$$\langle\pm,m|S_{0,z}|\pm,m\rangle = \pm\frac{1}{2}\cos\theta_m \equiv \pm\frac{1}{2}\alpha_m, \tag{S6a}$$

$$\langle +,m|S_{0,x}|+,m-1\rangle = +\frac{1}{2}\cos\frac{\theta_m}{2}\sin\frac{\theta_{m-1}}{2}, \tag{S6b}$$

$$\langle -,m|S_{0,x}|-,m-1\rangle = -\frac{1}{2}\sin\frac{\theta_m}{2}\cos\frac{\theta_{m-1}}{2}. \tag{S6c}$$

The diagonal matrix elements cause the frequency shift due to the coupling to the nuclear spins,which is $\sim|\bar{\mathbf{A}}_j|/2$, and the off-diagonal elements cause the coupling between neighboring energy levels $\sim|\bar{\mathbf{A}}_j|/4$. For a nuclear spin with relatively weak hyperfine coupling, namely,

$$|\bar{\mathbf{A}}_j|/2 \ll |g_{\rm e}\beta_{\rm e}B_0|/10, \tag{S7}$$

the hyperfine-induced mixing between different energy levels of the Bismuth center can be neglected. Then the interaction Hamiltonian becomes

$$H_{\rm hf} \approx \sum_{\eta=\pm}\sum_{m}\eta\alpha_m|\eta,m\rangle\langle\eta,m| \otimes \sum_j \mathbf{e}_z\cdot\bar{\mathbf{A}}_j\cdot\mathbf{I}_j, \tag{S8}$$

where

$$\alpha_m = \frac{g_{\rm e}\beta_{\rm e}B_0 + mA_{\rm Bi}}{\sqrt{A_{\rm Bi}^2(25-m^2)+(g_{\rm e}\beta_{\rm e}B_0+mA_{\rm Bi})^2}}, \tag{S9}$$

as defined in Eq. (S6a). This Hamiltonian is diagonal in the Bismuth center eigenstates, corresponding to the pure dephasing model (or secular approximation).

As will be demonstrated in Sec. , the contribution of a relatively strongly coupled ^{29}Si nuclear spin ($|\bar{\mathbf{A}}_j|/2 \gtrsim |g_{\rm e}\beta_{\rm e}B_0|/10$) to the ESEEM signal in our experiments is negligible. Thus the pure dephasing model in Eq. (S8) is justified.

The microwave pulses in general can induce many transitions in the Bi center as along as the transitions are within the bandwidth of the microwave cavity (164 kHz for the high-Q resonator). Since the interference between the transitions whose frequencies are not near-degenerate would cause oscillation much faster than the time resolution of the experiments and the

transitions with near-degenerate frequencies do not share an eigenstate of the Bi center (and therefore has no interference), we can reduce the Bi center to independent two-level systems ("fictitious spin-1/2") coupled to the Si spin bath.

The "fictitious spin-1/2" for the transition $|+,m\rangle \leftrightarrow |-,m-1\rangle$ has the Hamiltonian

$$H_m \equiv |+,m\rangle\langle +,m| \otimes H_m^+ + |-,m-1\rangle\langle -,m-1| \otimes H_m^-, \tag{S10}$$

with

$$H_m^+ = E_m^+ + \frac{1}{2}\alpha_m h_z + H_{\mathrm{Si}}, \tag{S11a}$$

$$H_m^- = E_{m-1}^- - \frac{1}{2}\alpha_{m-1} h_z + H_{\mathrm{Si}}, \tag{S11b}$$

where the Overhauser field along the z direction is

$$h_z \equiv \sum_j \mathbf{e}_z \cdot \bar{\mathbf{A}}_j \cdot \mathbf{I}_j.$$

The transition frequency or effective Larmor frequency of the fictitious spin-1/2

$$\omega_S = E_m^+ - E_{m-1}^- \approx 5A_{\mathrm{Bi}} + \frac{(2m-1)g_{\mathrm{e}}\beta_{\mathrm{e}}B_0}{10}.$$

Using a fictitious spin-1/2 to represent the transition $|-,m-1\rangle \leftrightarrow |+,m\rangle$ and define $\delta_m \equiv (\alpha_m - \alpha_{m-1})/2$ and $\bar{\alpha}_m \equiv (\alpha_m + \alpha_{m-1})/2$, we obtain the Hamiltonian

$$H_m = \omega_S S_z + \bar{\alpha}_m S_z h_z + \left(\frac{\delta_m}{2} h_z + H_{\mathrm{Si}}\right), \tag{S12}$$

which becomes Eq. 2 of main text if the bath has only one nuclear spin.

For a single nuclear spin (denoted as spin-j), the conditional Hamiltonian is

$$H_m^{\pm} = (\omega_I + \frac{\delta_m}{2}A_{j,zz})I_{j,z} + \frac{\delta_m}{2}A_{j,zx}I_{j,x} \pm \frac{\bar{\alpha}_m}{2}(A_{j,zz}I_{j,z} + A_{j,zx}I_{j,x}) \equiv \bar{H}_m \pm V_m. \tag{S13}$$

Note that we have dropped the ω_S-term (by working in the rotating reference frame) and for convenience chosen the coordinate system such that $A_{j,zy} = 0$. In the basis of the eigenstates of $\bar{H}_m$, the effective Hamiltonian can be written as

$$H_m^{\pm} = \tilde{\omega}_I I_{j,z} \pm \frac{1}{2}(AI_{j,z} + BI_{j,x}), \tag{S14}$$

where

$$\tilde{\omega}_I = \sqrt{(\omega_I + \delta_m A_{j,zz}/2)^2 + (\delta_m A_{j,zx}/2)^2},$$
$$A = \bar{\alpha}_m (A_{j,zz}\cos\theta + A_{j,zx}\sin\theta),$$
$$B = \bar{\alpha}_m (A_{j,zx}\cos\theta - A_{j,zz}\sin\theta),$$

with $\theta = \arcsin(\delta_m A_{j,zx}/2\tilde{\omega}_I)$.

Section S3 - Analytical solutions of ESEEM envelopes for the fictitious spin-1/2 model

In this Section, we provide details on the analytical solutions for the 2p-, 3p- and 5p-ESEEM (Kasumaj and Stoll, 2008), as a supplementary note on how the curves in Figs. 9-11 of Sec. VB in main text are calculated.

Section S3.1 - Formula

In the n-pulse ESEEM experiment, a sequence of n pulses $R_j \in \{R^x_{\pi/2}, R^y_{\pi/2}, R^x_\pi, R^y_\pi\}$ are applied at t_j ($j = 1, 2, \ldots n$), and then the signal $V_{n\mathrm{p}}$ is measured at the echo time t. In this Section, we take the pulses as ideal, i.e.,

$$R^{x/y}_\theta = \exp\left(-i\theta S_{x/y}\right).$$

With the pulse sequences described in Sec. VB of main text, the evolution for the 2p-, 3p-, and 5p-ESEEM is in turn

$$U_{2\mathrm{p}} = e^{-iH_m\tau} R^y_\pi e^{-iH_m\tau} R^x_{\pi/2}, \tag{S15a}$$

$$U_{3\mathrm{p}} = e^{-iH_m\tau} R^x_{\pi/2} e^{-iH_m T} R^x_{\pi/2} e^{-iH_m\tau} R^x_{\pi/2}, \tag{S15b}$$

$$U_{5\mathrm{p}} = e^{-iH_m\tau_2} R^y_\pi e^{-iH_m\tau_2} R^y_{\pi/2} e^{-iH_m T} R^y_{\pi/2} e^{-iH_m\tau_1} R^x_{\pi/2} e^{-iH_m\tau_1} R^x_{\pi/2}. \tag{S15c}$$

We assume the Bi center is initially in the state $\rho_S = |-, m-1\rangle\langle -, m-1|$ and the nuclear spin bath is in the maximally mixed state ρ_{B}. The spin coherence at the echo time is

$$V_{2\mathrm{p}/3\mathrm{p}/5\mathrm{p}} = 2\mathrm{Tr}\left[S_{0,x} U_{2\mathrm{p}/3\mathrm{p}/5\mathrm{p}} \left(\rho_S \otimes \rho_{\mathrm{B}}\right) U^\dagger_{2\mathrm{p}/3\mathrm{p}/5\mathrm{p}}\right]. \tag{S16}$$

Section S3.2 - Exact formula for a single nuclear spin

For 2p-ESEEM, the modulation amplitude due to the j-th ^{29}Si spin is

$$V_{2\mathrm{p},j}(\tau) = 1 - \frac{k_j}{4}\left[2 - 2\cos\left(\omega_\uparrow\tau\right) - 2\cos\left(\omega_\downarrow\tau\right) + \cos\left(\omega_\uparrow\tau - \omega_\downarrow\tau\right) + \cos\left(\omega_\uparrow\tau + \omega_\downarrow\tau\right)\right], \tag{S17}$$

where $k_j = \left(\frac{B\tilde{\omega}_I}{\omega_\uparrow\omega_\downarrow}\right)^2$, and (the same as Eq. 6 of main text)

$$\omega_\uparrow \equiv \sqrt{(\tilde{\omega}_I + \frac{A}{2})^2 + \frac{B^2}{4}}, \quad \omega_\downarrow \equiv \sqrt{(\tilde{\omega}_I - \frac{A}{2})^2 + \frac{B^2}{4}}.$$

For the 3p-ESEEM, the modulation amplitude is given by

$$\begin{aligned} V_{3\mathrm{p},j}(\tau, T) &= \frac{1}{2}\left[V^\uparrow_{3\mathrm{p},j}(\tau, T) + V^\downarrow_{3\mathrm{p},j}(\tau, T)\right] \\ &= 1 - \left\{\frac{k_j}{4}\left[1 - \cos(\omega_\downarrow\tau)\right]\left[1 - \cos(\omega_\uparrow T + \omega_\uparrow\tau)\right] + \left[\omega_\uparrow \leftrightarrow \omega_\downarrow\right]\right\}. \end{aligned} \tag{S18}$$

where

$$V^\uparrow_{3\mathrm{p},j}(\tau, T) = 1 - \frac{k_j}{2}\left[1 - \cos(\omega_\downarrow\tau)\right]\left[1 - \cos(\omega_\uparrow T + \omega_\uparrow\tau)\right]$$

and $V^\downarrow_{3\mathrm{p},j}(\tau, T)$ is obtained by exchanging $\uparrow$ and $\downarrow$.

For the 5p-ESEEM, the modulation amplitude is

$$V_{5\mathrm{p},j}(\tau_1, \tau_2, T) = \frac{1}{4}\left(V^{\uparrow,+}_{5\mathrm{p},j} - V^{\uparrow,-}_{5\mathrm{p},j} + V^{\downarrow,+}_{5\mathrm{p},j} - V^{\downarrow,-}_{5\mathrm{p},j}\right), \tag{S19}$$

where

$$\begin{aligned} V^{\uparrow,\pm}_{5\mathrm{p},j} =& V_{2\mathrm{p},j}\left(\tau_1\right) V_{2\mathrm{p},j}\left(\tau_2\right) \pm b_{5\mathrm{p},j}\left[4k_j^2 C^\uparrow_j - 2k_j^2\cos\phi_{\downarrow,-}\cos\left(T\omega_\uparrow + \phi_{\uparrow,+}\right)\right. \\ &\left. -4k_j\cos^4\eta_j\cos\left(T\omega_\uparrow + \phi_{\uparrow,+} + \phi_{\downarrow,+}\right) - 4k_j\sin^4\eta_j\cos\left(T\omega_\uparrow + \phi_{\uparrow,+} - \phi_{\downarrow,+}\right)\right], \end{aligned} \tag{S20}$$

and $V_{5\text{p},j}^{\downarrow,\pm}$ is obtained from the expression above by exchanging $\uparrow$ and $\downarrow$, with

$$\begin{aligned}
\phi_{\uparrow/\downarrow,\pm} &= (\tau_1 \pm \tau_2)\,\omega_{\uparrow/\downarrow}/2,\\
\cos^2\eta_j &= \left[\tilde{\omega}_I^2 - \frac{1}{4}(\omega_\uparrow - \omega_\downarrow)^2\right]/\left(\omega_\uparrow\omega_\downarrow\right),\\
\sin^2\eta_j &= \left[\frac{1}{4}(\omega_\uparrow + \omega_\downarrow)^2 - \tilde{\omega}_I^2\right]/\left(\omega_\uparrow\omega_\downarrow\right),\\
C_j^\uparrow &= \cos\left(\frac{\tau_1\omega_\uparrow}{2}\right)\cos\left(\frac{\tau_2\omega_\uparrow}{2}\right)\sin\left(\frac{\tau_1\omega_\downarrow}{2}\right)\sin\left(\frac{\tau_2\omega_\downarrow}{2}\right),\\
b_{5\text{p},j} &= \sin\left(\frac{\tau_1\omega_\uparrow}{2}\right)\sin\left(\frac{\tau_2\omega_\uparrow}{2}\right)\sin\left(\frac{\tau_1\omega_\downarrow}{2}\right)\sin\left(\frac{\tau_2\omega_\downarrow}{2}\right).
\end{aligned}$$

Section S3.3 - Contributions of multiple nuclear spins

For multiple nuclear spins, if they are taken as independent (with interactions between the nuclear spins neglected), the ESEEM signal is obtained by applying the product rule for each pathway followed by average over different pathways ((Kasumaj and Stoll, 2008)). For the 2p/3p/5p-ESEEM signals are in turn

$$V_{2\text{p}} = \prod_j V_{2\text{p},j}, \tag{S21a}$$

$$V_{3\text{p}} = \frac{1}{2}\left(\prod_j V_{3\text{p},j}^{\uparrow} + \prod_j V_{3\text{p},j}^{\downarrow}\right), \tag{S21b}$$

$$V_{5\text{p}} = \frac{1}{4}\left(\prod_j V_{5\text{p},j}^{\uparrow,+} - \prod_j V_{5\text{p},j}^{\uparrow,-} + \prod_j V_{5\text{p},j}^{\downarrow,+} - \prod_j V_{5\text{p},j}^{\downarrow,-}\right). \tag{S21c}$$

Above we have considered the ESEEM signal due to the transition $|-,m-1\rangle \leftrightarrow |+,m\rangle$. The transitions $|-,m\rangle \leftrightarrow |+,m-1\rangle$ can be considered similarly. We use $V_{2\text{p}/3\text{p}/5\text{p}}^{(m,\pm)}$ to denote the contribution to the 2p/3p/5p-ESEEM signal by the transition $|-,m\rangle \leftrightarrow |+,m\pm 1\rangle$ [with a superscript index $(m,\pm)$ attached to the signals in Eq. (S21)].

Section S3.4 - Weighting factors of different Bi spin transitions

To take into account the contributions of all possible transitions, we assume the Bi center spin is initially randomly populated in the lower manifold of the eigenstates, i.e.,

$$\rho_{\text{Bi}} = \sum_{m=-4}^{+4} P_m|-,m\rangle\langle -,m|,$$

with the probabilities $\sum_m P_m = 1$. The coherence of different transitions is summed up as

$$V_{2\text{p}/3\text{p}/5\text{p}} = \sum_{m,\pm} W_{m,\pm} V_{2\text{p}/3\text{p}/5\text{p}}^{(m,\pm)}, \tag{S22}$$

with the weighting factor $W_{m,\pm}$ accounting for the initial probabilities P_m and the different amplitudes for different transitions given the microwave pulse spectra and the distribution of the hyperfine coupling A_{Bi} due to the strain in the Si layer.

To determine the relative transition weights, we resort to a complete model of the experiment, based on the physical parameters, namely the control pulse amplitude and temporal profile, the Rabi frequencies distribution, and the repetition time Γ_{rep}^{-1}. We use a simulation code that was purposely written. For each class of fictitious spin-1/2's with given Larmor frequency ω_S and Rabi frequency Ω_R, the program integrates the Bloch equations to compute the time dependence of the spin density matrix $\rho(t)$. The initial conditions take into account the Rabi-frequency-dependent spin relaxation T_1 because of the Purcell effect, by taking $\rho(0) = |x\rangle\langle x|$ (the spin polarized along the x-axis). The simulation results are furthermore averaged over the strain-induced distribution of the ^{209}Bi hyperfine coupling $\sigma_A(A_{\text{Bi}})$, which results in the distribution of the transition frequency $\sigma_S(\omega_S)$, and over the distribution of the Rabi frequency $\sigma_R(\Omega_R)$. The Bi hyperfine coupling distribution $\sigma_A(A_{\text{Bi}})$ is taken to be flat since the inhomogeneous broadening (∼50 MHz) is two orders of magnitude larger than the cavity bandwidth (∼160 kHz).

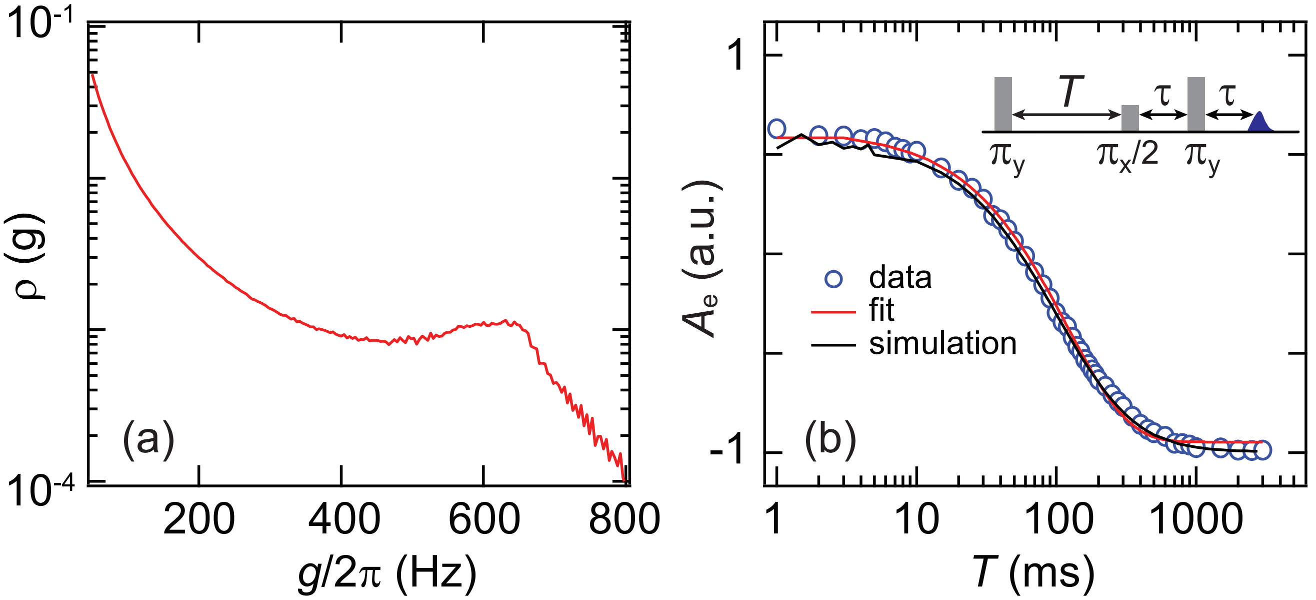

Figure S1. Inhomogeneous coupling strength. (a) The normalized density of spins $\rho(g)$ versus the spin-photon coupling strength for the transition $|-,4\rangle \rightarrow |+,5\rangle$. (b) Inversion recovery of spin polarization measured (in symbols) at a repetition rate of 0.2 Hz. The fit yields a $T_1 \approx 120$ ms. The simulation incorporating 18 transitions and $\rho(g)$ is consistent with the data for only a suitable pulse amplitude.

Table S1. The relative transition weights $W_{m,\pm}$ from the simulation.

m	4	3	2	1	0	-1	-2	-3	-4
$\lvert -,m\rangle \leftrightarrow \lvert +,m+1\rangle$	0.1075	0.0977	0.0909	0.0766	0.0586	0.0326	0.0204	0.0086	0.007
$\lvert -,m\rangle \leftrightarrow \lvert +,m-1\rangle$	0.007	0.0086	0.0204	0.0326	0.0586	0.0766	0.0909	0.0977	0.1075

The Rabi frequency distribution $\sigma_R(\Omega_R)$ is computed using finite-element modelling of the AC field spatial profile generated by running a constant current through the resonator inductance wire. The AC field map and the resulting distribution $\sigma_R(\Omega_R)$ are shown in Fig. S1. To calibrate the AC pulse amplitude, we rely on the fact that due to the AC field inhomogeneity and to the Purcell spin relaxation, the measured T_1 in an inversion recovery sequence is in fact amplitude-dependent. We then adjust the pulse amplitude in the simulation so that the simulated inversion recovery sequence reproduces the same relaxation curve as measured. The weighting factors thus derived from simulating the experimental data are given in Table. S1.

Section S3.5 - Comparison with experiments

For comparison with experimental data, the curves in Figs. 9-11 of main text are obtained by first calculating $V_{2p/3p/5p}$ using Eq. (S22) for each nuclear spin configuration (with random positions of the ^{29}Si nuclei) and then averaging over different nuclear spin spatial configurations.

Section S4 - Justification of independent bath spin approximation

In the calculations above, the nuclear spins in the bath are taken as independent, i.e., the interactions between the ^{29}Si spins are neglected. In this Section, we will provide a justification of the approximation by calculating the Bi spin coherence with the cluster correlation expansion (CCE) (Yang and Liu, 2008, 2009; Zhao et al., 2012). The leading order of expansion (CCE-1) corresponds to the independent bath spin approximation used in the previous Section. The numerical calculation shows that the CCE-1 is a good approximation for the timescale relevant to the experimental studies of the ESEEM.

We check the contributions of various orders of the nuclear spin correlations to the ESEEM signals using the fictitious spin-1/2 model in Eq. (S10) and the CCE method (Yang and Liu, 2008, 2009; Zhao et al., 2012). The interactions between the ^{29}Si

nuclear spins are included in $H_{\rm Si}$. The CCE method allows us to consider in a recursive way the dynamics of the nuclear spin bath due to correlations of different sizes (CCE-n for n-spin irreducible correlation).

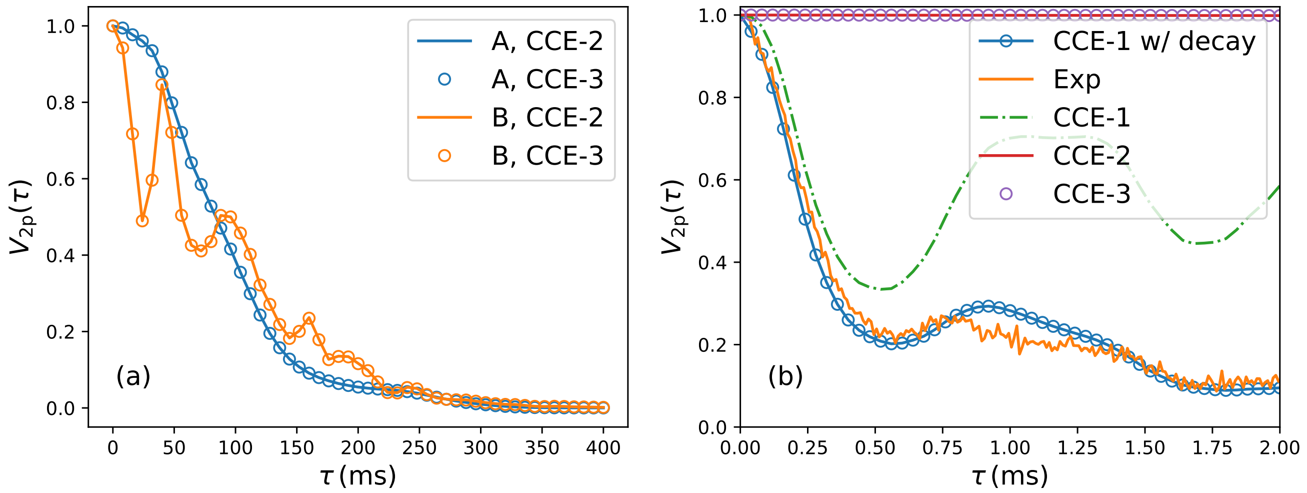

Figure S2. CCE calculation of the Hahn echo signal of a Bi donor spin coupled to a ^{29}Si nuclear spin bath. The CCE-2 and CCE-3 results have excluded the CCE-1 effects. (a) Decoherence for two different random bath configurations (denoted as “A” and “B”), calculated up to CCE-2 or CCE-3 (both with CCE-1 contributions excluded). $B_0 = 1$ G. The number of bath spins $N = 2000$. The negligible difference between CCE-2 and CCE-3 indicates that the CCE-2 has already converged. (b) Comparison of the CCE results with the experimental ESEEM signal for $B = 1$ G. For the “CCE-1 w/ decay”, an overall exponential decay is included as explained in the main text.

Considering the 2p-ESEEM for example, the spin coherence is expanded as

$$V_{2p} = \prod_{C} \tilde{V}_{2p}^{C}, \tag{S23}$$

with $\tilde{V}_{2p}^{C}$ defined as the irreduible correlation of cluster C excluding the irreducible correlations of all sub-clusters, i.e.,

$$\tilde{V}_{2p}^{C} \equiv \frac{V_{2p}^{C}}{\prod_{C' \subset C} \tilde{V}_{2p}^{C'}}, \tag{S24}$$

where V_{2p}^{C} is the center spin coherence under coupling to the cluster C in the spin bath (the bath spins outside the cluster dropped).

As shown in Fig. S2, in the timescale relevant to the ESEEM signals in the experiments, the echo is affected mainly by the single-spin dynamics (CCE-1) and the contributions from pair dynamics (CCE-2) and higher order correlations in the bath are negligible. The decoherence due to CCE-2 and CCE-3 occurs at timescales of ~ 100 ms [Fig. S2(a)], which are much longer than the experimental time regime (~ 1 ms).

Thus it is well justified to take the nuclear spins in the bath as independent of each other.

Section S5 - Effect of a strongly coupled Si-29 nuclear spin

In this Section, we show that the strongly coupled ^{29}Si nuclear spins have negligible contributions to the ESEEM signals. Therefore the calculated ESEEM signals presented in Figs. 9-11 in the main text are those from ^{29}Si with hyperfine couplings < 20 kHz.

In the calculations, we have assumed the pure dephasing model (the secular approximation) in which the transitions between different eigenstates of the Bi center spin due to the hyperfine coupling to the ^{29} nuclear spins are taken as negligible. This approximation is well justified if the hyperfine coupling is much less than the energy splitting between different Bi center spin states [Eq. (S7)]. When the coupling is strong the secular and the CCE-1 approximation become invalid. Using simulations that take into account exactly the effect of the strongly coupled nuclear spin, we show that a strongly coupled spin has negligible

effects on the ESEEM signal in the timescale considered in the experiments. A strongly coupled spin contributes only fast oscillations in the signal, which would vanish if we take into account the inhomogeneous broadening effects. In addition, the influence of the strongly coupled spins on the distant weakly coupled spins are also negligible.

For a ^{29}Si with hyperfine coupling $\gtrsim 200$ kHz, the state mixing due to the hyperfine coupling enables many transitions and the interference between these transitions will cause rather complicated and fast oscillations in the spin echo signal. This is seen in Figs. S7. However, in such a strong coupling case, the ESEEM frequencies depend sensitively on the local Overhauser field on the Bi electron spin. Since the Overhauser field has a large inhomogeneous broadening (~ 0.5 MHz), the ensemble average over the nuclear spin state thermal distribution leads to a rapid decay of the signal (decay in $< 1\ \mu$s). We estimate that about 10% Bi centers have one or more ^{29}Si with coupling > 200 kHz in the proximity, which would contribute to a fast initial decay of the total echo signal in $< 1\ \mu$s by about 10%. In the experiments, the echo signal is measured at times much greater than μs (the first time point is $\sim 1\,\mathrm{ms}$). As shown in Figs. S5 and S7, the ESEEM amplitude of a nuclear spin with coupling strength between 20 kHz and 200 kHz is much less than 1%. And for nuclear spins with the relatively weak hyperfine coupling $(2\pi)^{-1}|\bar{\mathbf{A}}_j| < 100$ kHz, the pure dephasing model produces results with negligible errors of the modulation frequencies from the exact solution (Figs. S5-S6). Furthermore, the systematic numerical studies (Fig. S9) show that a nearby Si nuclear spin with coupling < 200 kHz has negligible effects on the ESEEM due to other distant nuclear spins.

Considering these different contributions of Si nuclear spins of different hyperfine couplings, it is justified to assume the pure-dephasing model and consider only the contributions of those Si nuclear spins that have couplings weaker than a certain cut-off (chosen as $|\bar{\mathbf{A}}_j| \leq 20$ kHz).

Section S5.1 - CCE-1 for the multi-level central system

Strong hyperfine interaction would cause the mixing between the Bismuth center eigenstates. To consider the effect of a strongly coupled ^{29}Si nuclear spin, we take the electron spin $\mathbf{S}_0$, the ^{209}Bi nuclear spin $\mathbf{I}_0$, and the j_0 "strongly coupled" ^{29}Si nuclear spins (denoted as $\mathbf{I}_j$ for $1 \leq j \leq j_0$) as a hybrid center spin system. The hybrid center system can be diagonalized, with $2 \times 10 \times 2^{j_0}$ eigenstates, separated into two manifold $\{|\pm, m\rangle\}$. The corresponding eigenenergies of the hybrid center spin system is denoted as $E_m^{\pm}$. See Fig. S3 for an example of the eigenenergies of a hybrid spin system with one strongly coupled ^{29}Si nuclear spin.

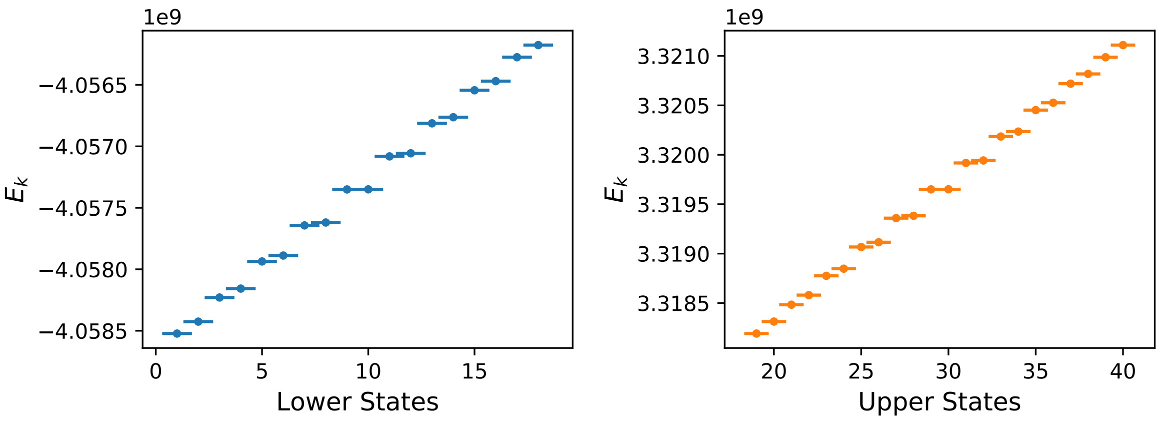

Figure S3. Eigenenergies of a hybrid spin system composed of the Bi donor electron spin $\mathbf{S}_0$, the ^{209}Bi nuclear spin $\mathbf{I}_0$, and a "strongly coupled" ^{29}Si nuclear spin at position $\mathbf{r}_1 = (1.8, 0.4, -0.9)$ nm. The Fermi-contact hyperfine coupling to the Si spin is $A_{\mathrm{fc},1}/(2\pi) \approx 250$ kHz. The magnetic field along the z-axis $B_0 = 1$ G. The x axis denotes the state label for the upper and lower eigenstates (the $\pm$ manifolds).

The Hamiltonian of the hybrid center system plus the rest $N - j_0$ weakly coupled ^{29}Si nuclear spins can be written as

$$H = \sum_{\eta=\pm} \sum_{m} E_m^{\eta} |\eta, m\rangle\langle\eta, m| + \sum_{j=j_0+1}^{N} \left(\mathbf{S}_0 \cdot \bar{\mathbf{A}}_j \cdot \mathbf{I}_j + g_n \beta_n \mathbf{B}_0 \cdot \mathbf{I}_j\right), \tag{S25}$$

where the dipole-dipole interaction between the nuclear spins in the bath is neglected because it is weak and would have effects only on higher order CCE. By assumption, the hyperfine couplings $|\bar{\mathbf{A}}_j|$ (for $j > j_0$) are much smaller than the energy differences among the eigenstates of the central system (otherwise they would have been absorbed into the hybrid central

system). Therefore, we have the pure dephasing model with the bath Hamiltonians conditioned on the states of the central system (for the conciseness, we label (η, m) as k)

$$H^{(k)} \equiv H'_{\mathrm{Si}} + \langle k|H_{\mathrm{hf}}|k\rangle = H'_{\mathrm{Si}} + \eta\alpha_m \sum_j \mathbf{A}_j \cdot \mathbf{I}_j, \tag{S26}$$

where the Si nuclear spin bath Hamiltonian H'_{Si} excludes the strongly coupled ^{29}Si nuclear spins $\mathbf{I}_j$ (for $j \le j_0$) and neglects the dipolar interactions between the Si nuclear spins.

In the echo experiment, a sequence of n pulses

$$R_j \in \{R^x_{\pi/2}, R^x_\pi, R^y_{\pi/2}, R^y_\pi\}$$

are applied at t_j $(j = 1,2,\ldots n)$, and then the signal $V_{3\mathrm{p}}$ is measured at time t. The $\pi/2$ and π control pulses are taken as $R^{x/y}_{\pi/2} \equiv e^{-i\left(\Omega_{\mathrm{R}} S_{0,x/y} + H_{\mathrm{Bi}}\right)\tau_{\mathrm{p}}/2}$ and $R^{x/y}_{\pi} = e^{-i\left(2\Omega_{\mathrm{R}} S_{0,x/y} + H_{\mathrm{Bi}}\right)\tau_{\mathrm{p}}/2}$, where τ_{p} is the duration of a π pulse and Ω_{R} the Rabi frequency. Note that the pulse durations are assumed much shorter than the timescales of dynamics of the ^{29}Si bath spins and thus the rotation transform does not include H'_{Si}. If the system starts with a certain state $|k_0\rangle \otimes |J\rangle$ at $t = 0$ (in which $|k_0\rangle$ denotes a certain eigenstate $|-,m\rangle$ and $|J\rangle$ a certain bath state), the state at time t is

$$\sum_{k_1,k_2,\ldots,k_n} C_{k_0,k_1,\ldots k_n} e^{-i\phi_{k_1,k_2,\ldots k_n}} |k_n\rangle \, |J_{k_1,k_2,\ldots k_n}\rangle\,,$$

where $|k_j\rangle$ denotes an eigenstate $|\pm, m\rangle$, the phase

$$\phi_{\mathbf{k}} = E_{k_1} t_1 + E_{k_2}(t_2 - t_1) + \cdots + E_{k_n}(t - t_n),$$

the coefficients of the eigenstates

$$C_{k_0,\mathbf{k}} \equiv \langle k_n|R_n|k_{n-1}\rangle \cdots \langle k_2|R_2|k_1\rangle\langle k_1|R_1|k_0\rangle,$$

and the bath state

$$|J_{\mathbf{k}}\rangle \equiv e^{-iH^{(k_n)}(t-t_n)} \cdots e^{-iH^{(k_2)}(t_2-t_1)} e^{-iH^{(k_1)}t_1}|J\rangle,$$

with the shorthand notation $\mathbf{k} = (k_1, k_2, \ldots, k_n)$. The echo signal is

$$V_{2\mathrm{p}/3\mathrm{p}/5\mathrm{p}} \equiv \langle S_{0,x}(t)\rangle = \sum_{\mathbf{k},\mathbf{k}'} C^*_{k_0,\mathbf{k}'} C_{k_0,\mathbf{k}} e^{i\phi_{\mathbf{k}'} - i\phi_{\mathbf{k}}} \langle k'_n|S_{0,x}|k_n\rangle \, \langle J_{\mathbf{k}'} \,|J_{\mathbf{k}}\rangle\,. \tag{S27}$$

In general, the nuclear spin state overlap $\langle J_{\mathbf{k}'} \,|J_{\mathbf{k}}\rangle$ is computed using the CCE (Yang and Liu, 2008, 2009; Zhao et al., 2012), and in our current case for the Hamiltonian in Eq. (S25), CCE-1 gives the exact solution. The result of $V_{2\mathrm{p}/3\mathrm{p}/5\mathrm{p}}$ is further averaged over different initial states $|k_0\rangle$ and $|J\rangle$ in the thermal distribution.

Without loss of generality we consider the 3p-ESEEM experiment with the magnetic field $B_0 = 1$ G. For the 3p-ESEEM, the CCE-1 result is

$$\begin{aligned} V_{3\mathrm{p}} = &\sum_{k_1,k_2,k_3,k'_1,k'_2,k'_3} C^*_{k_0,k'_1,k'_2,k'_3} C_{k_0,k_1,k_2,k_3} \exp\left(i(\phi^{(k'_1,k'_2,k'_3)} - \phi^{(k_1,k_2,k_3)})\right) \\ &\langle k'_3|S_{0,x}|k_3\rangle \left| \prod_{j=j_0+1}^{N} \mathrm{Tr}\left[\frac{1}{2} U_j^{(k'_1,k'_2,k'_3)\dagger} U_j^{(k_1,k_2,k_3)}\right] \right|, \end{aligned} \tag{S28}$$

where $U_j^{(k_1,k_2,k_3)}$ is the evolution operator of the spin $\mathbf{I}_j$ for the center spin pathway $k_0 \to k_1 \to k_2 \to k_3$.

For the hybrid center that contains only the Bi electron and nuclear spins (no ^{29}Si spins), the nonzero elements of the $S_{0,x}$ operator are

$$\begin{aligned}
\langle +,m|S_{0,x}|-,m-1\rangle &= +\frac{1}{2}\cos\frac{\theta_m}{2}\cos\frac{\theta_{m-1}}{2}, \\
\langle -,m|S_{0,x}|+,m-1\rangle &= -\frac{1}{2}\sin\frac{\theta_m}{2}\sin\frac{\theta_{m-1}}{2}, \\
\langle +,m|S_{0,x}|+,m-1\rangle &= +\frac{1}{2}\cos\frac{\theta_m}{2}\sin\frac{\theta_{m-1}}{2}, \\
\langle -,m|S_{0,x}|-,m-1\rangle &= -\frac{1}{2}\sin\frac{\theta_m}{2}\cos\frac{\theta_{m-1}}{2}.
\end{aligned}$$

Table S2. Absolute values of the matrix elements of $S_{0,x}$ in the basis $\{|\eta, m\rangle\}$ for $B_0 = 1$ G.

$\|-,4\rangle$	0	0.14	0	0	0	0	0	0	0	0	0	0	0	0	0	0	0	0.07	0	0.47
$\|-,3\rangle$	0.14	0	0.19	0	0	0	0	0	0	0	0	0	0	0	0	0	0.12	0	0.42	0
$\|-,2\rangle$	0	0.19	0	0.21	0	0	0	0	0	0	0	0	0	0	0	0.17	0	0.37	0	0
$\|-,1\rangle$	0	0	0.21	0	0.22	0	0	0	0	0	0	0	0	0	0.22	0	0.32	0	0	0
$\|-,0\rangle$	0	0	0	0.22	0	0.22	0	0	0	0	0	0	0	0.27	0	0.27	0	0	0	0
$\|-,-1\rangle$	0	0	0	0	0.22	0	0.21	0	0	0	0	0	0.32	0	0.22	0	0	0	0	0
$\|-,-2\rangle$	0	0	0	0	0	0.21	0	0.19	0	0	0	0.37	0	0.17	0	0	0	0	0	0
$\|-,-3\rangle$	0	0	0	0	0	0	0.19	0	0.14	0	0.42	0	0.12	0	0	0	0	0	0	0
$\|-,-4\rangle$	0	0	0	0	0	0	0	0.14	0	0.47	0	0.07	0	0	0	0	0	0	0	0
$\|+,-5\rangle$	0	0	0	0	0	0	0	0	0.47	0	0.16	0	0	0	0	0	0	0	0	0
$\|+,-4\rangle$	0	0	0	0	0	0	0	0.42	0	0.16	0	0.21	0	0	0	0	0	0	0	0
$\|+,-3\rangle$	0	0	0	0	0	0	0.37	0	0.07	0	0.21	0	0.24	0	0	0	0	0	0	0
$\|+,-2\rangle$	0	0	0	0	0	0.32	0	0.12	0	0	0	0.24	0	0.26	0	0	0	0	0	0
$\|+,-1\rangle$	0	0	0	0	0.27	0	0.17	0	0	0	0	0	0.26	0	0.27	0	0	0	0	0
$\|+,0\rangle$	0	0	0	0.22	0	0.22	0	0	0	0	0	0	0	0.27	0	0.27	0	0	0	0
$\|+,1\rangle$	0	0	0.17	0	0.27	0	0	0	0	0	0	0	0	0	0.27	0	0.26	0	0	0
$\|+,2\rangle$	0	0.12	0	0.32	0	0	0	0	0	0	0	0	0	0	0	0.26	0	0.24	0	0
$\|+,3\rangle$	0.07	0	0.37	0	0	0	0	0	0	0	0	0	0	0	0	0	0.24	0	0.21	0
$\|+,4\rangle$	0	0.42	0	0	0	0	0	0	0	0	0	0	0	0	0	0	0	0.21	0	0.16
$\|+,5\rangle$	0.47	0	0	0	0	0	0	0	0	0	0	0	0	0	0	0	0	0	0.16	0

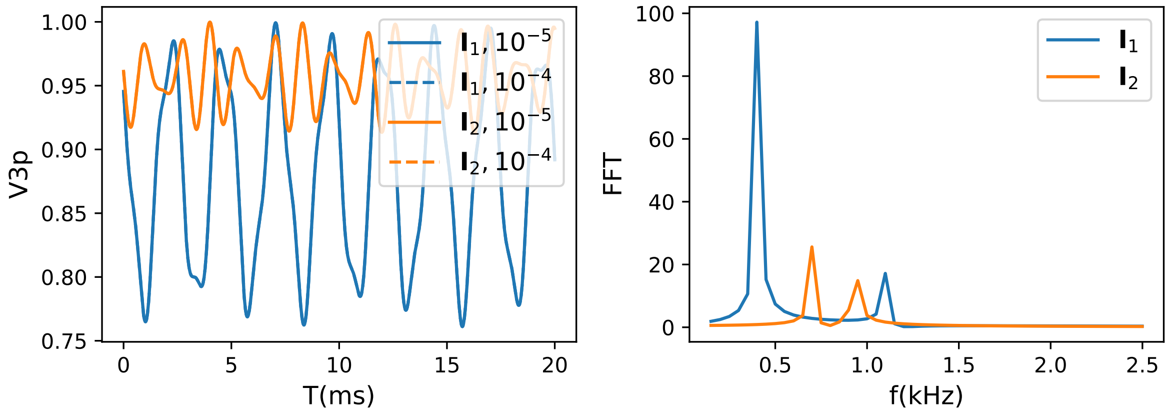

Figure S4. Exact solution of the 3p-ESEEM of a Bi donor spin coupled to only one ^{29}Si nuclear spin. The Fermi contact and dipolar hyperfine couplings are $A_{\mathrm{cf},1} = 2.4$ kHz and $A_{\mathrm{dd},1} = 0.6$ kHz in the first case (labeled as $\mathbf{I}_1$), and $A_{\mathrm{cf},2} = 0.8$ kHz and $A_{\mathrm{dd},2} = 0.5$ kHz in the second case (labeled as $\mathbf{I}_2$). The external field $B_0 = 1$ G. The left panel shows the ESEEM, and the right panel shows the Fourier transform of the ESEEM. The cutoff thresholds $|C^*_{\mathbf{k}'}C_{\mathbf{k}}| < 10^{-5}$ and 10^{-4} for a pathway to be dropped are indicated in the left panel. The two choices of cutoff thresholds produce nearly identical results.

See numerical values in Table. S2.

Not all of the pathways have contributions to the ESEEM signal because of the inhomogeneous broadening and selection rules. For instance, considering a hybrid center spin system that contains only the Bi electron and nuclear spins, the phase difference accumulated for $k_j = (+, m)$ and $k'_j = (+, m')$ during time from t_{j-1} to t_j is

$$\phi_{k'_j} - \phi_{k_j} = (E^+_{m'} - E^+_m) \cdot (t_j - t_{j-1}) \approx \frac{m' - m}{10}(g_e \beta_e B_0 + h_z) \cdot (t_j - t_{j-1}), \tag{S29}$$

where h_z is the Overhauser field from the bath spins. For the relevant timescales (~ 1 ms) and inhomogeneous broadening of h_z (which is ~ 0.5 MHz), the ensemble averaged phase factor would vanish unless $k_j = k'_j$. In the numerical simulation, a pathway $(\mathbf{k}', \mathbf{k})$ is dropped if (1) the echo condition

$$\phi_{\mathbf{k}} - \phi_{\mathbf{k}'} = 0, \tag{S30}$$

is not satisfied, or (2) the amplitude is too small, e.g., $|C^*_{\mathbf{k}'}C_{\mathbf{k}}| < 10^{-4}$ (see Fig. S4).

Section S5.2 - Exact simulation for one ^{29}Si spin

Taking the special case of Eq. (S28) for $j_0 = 1$ and $N = 1$, we can obtain the exact solution of the ESEEM due to a single ^{29}Si spin. The result for a certain initial state $|k_0\rangle$ is

$$V_{3p} = \sum_{k_1,k_2,k_3,k_1',k_2',k_3'} C^*_{k_0',k_1',k_2',k_3} C_{k_0,k_1,k_2,k_3} \langle k_3'|S_{0,x}|k_3\rangle \exp\left(i\phi^{(k_1',k_2',k_3')} - i\phi^{(k_1,k_2,k_3)}\right), \tag{S31}$$

where $\phi^{(k_1,k_2,k_3)} \equiv (E_{k_1} + E_{k_3})\tau + E_{k_2}T$ is the phase accumulated for the pathway $k_0 \to k_1 \to k_2 \to k_3$.

In numerical simulations we neglect the pathways that have negligible probabilities $C^*_{k_0',k_1',k_2',k_3} C_{k_0,k_1,k_2,k_3}|$. For instance, Fig. S4 compares the results with pathways neglected when $|C^*_{\mathbf{k}'} C_{\mathbf{k}}| < 10^{-5}$ or 10^{-4}. Actually, for these two cases shown in the figure, there are only 4 main contributing pathways, since most pathways have no contributions in the ESEEM signal after taking into account of the inhomogeneous broadening effects (see Eq. S29).

Section S5.3 - Justification of CCE-1 approximation for weakly coupled bath spins

Now we compare the exact solution and CCE-1 approximation for a single ^{29}Si bath spins ($N = 1$). We use Eq. (S31) for the exact solution and Eq. (S28) with $j_0 = 0$ and $N = 1$ for the CCE-1 approximation. The pathways of the Bi spin that do not satisfy the echo condition [Eq. (S30)] are not included in the simulation.

We expect that when the hyperfine coupling is comparable to the level splitting of the Bismuth donor spin within each manifold, the secular and the CCE-1 approximation become invalid (Eq. S7). Figure S5 compares the exact solution and the CCE-1 approximation for various hyperfine coupling strengths. As expected, the CCE-1 agrees well with the exact solution for relatively weak coupling. The deviation of the ESEEM frequency calculated by the CCE-1 from the exact solution is shown in Fig. S6 as a function of the hyperfine coupling strength. For coupling weaker than 20 kHz, the error is less than 5%. Furthermore, the ESEEM depth (shown in Fig. S6) becomes negligible for coupling greater than 20 kHz for the field. (It should be noted that if the echo condition in Eq. (S30) is applied, strong hyperfine coupling would lead to mixing between Bi spin states, causing fast oscillations with amplitude increasing with the coupling strength. Such fast oscillations, however, decay rapidly to zero due to inhomogeneous broadening. See Sec. below for more discussions.)

Considering both the ESEEM frequency calculation precision and the modulation depth, a nuclear spin with hyperfine coupling weaker than 20 kHz can be well approximated by the CCE-1.

Section S5.4 - ESEEM due to a strongly coupled ^{29}Si spin

Figure. S7 show the exact simulation of the 3p-ESEEM due to a strongly coupled ^{29}Si spin. Two coupling strengths ($\sim$ 100 kHz and $\sim$ 200 kHz) are considered. The strong hyperfine coupling induces mixing between the Bi spin states (violating the pure dephasing approximation). To show the effect of state mixing, we do not impose the echo condition Eq. (S30), and therefore need to include about 100 pathways to produce converged results. The interference between different pathways (due to the mixing among Bi spin states) induce fast and complicated modulations. The Fourier transform shows that the ESEEM frequencies are spread around 280 kHz, which is approximately the splitting between Bi levels in absence of Si spins. The fact that modulation frequency is near 280 kHz confirms that the ESEEM is mostly due to the mixing of the Bi spin states. On the contrary, in the case of weakly coupled ^{29}Si spins where the mixing between different Bi levels is negligible, the ESEEM is due to the mixing between different ^{29}Si nuclear spin states, so there the ESEEM has frequencies essentially determined by the ^{29}Si nuclear spin Larmor frequency (see Fig. S5).

The rapid oscillations due to interference between different Bi spin states, however, have their frequencies sensitively depending on the external field and the Overhauser field due to the random configurations of the nuclear spin baths (the inhomogeneous broadening). Therefore, the ensemble average over the distribution of the Overhauser field h_z (which has a broadening of about 0.5 MHz) would lead to rapid decay of the modulation (in μs timescales). This is indeed shown in Fig. S8. Thus, the contributions of the strongly coupled nuclear spins are not observable in the timescales relevant in the experiments ($\sim$ms).

Section S5.5 - Influence of a strongly coupled ^{29}Si spin on weakly coupled ^{29}Si spins

In general, a strongly coupled nuclear spin may affect the ESEEM due to a weakly coupled nuclear spin. This is because the Bi spin states can be mixed by the strong coupling. The state mixing renormalizes the effective couplings between the weakly coupled nuclear spin and the "ficticious" spin-1/2 of the Bi donor [in particular, α_m and α_{m-1} in Eq. (S13)] and hence affects the ESEEM. To exam such an effect, we calculate the ESEEM due to a weakly coupled nuclear spin $\mathbf{I}_2$ in the presence or in the absence of a strongly coupled nuclear spin $\mathbf{I}_1$. The weakly coupled nuclear spin is considered using the CCE-1 and the strongly

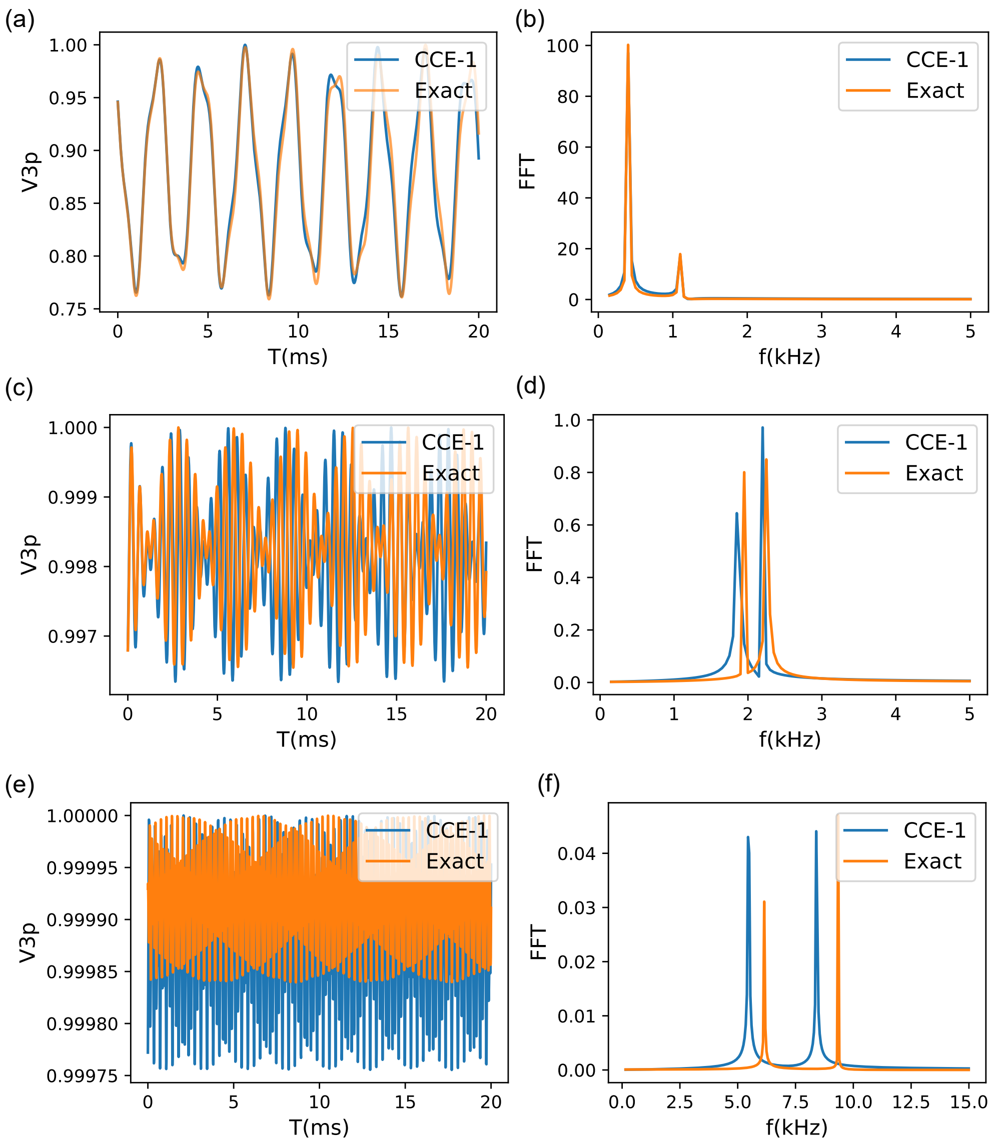

Figure S5. Comparison between exact solution [Eq. (S31)] and the CCE-1 approximation [Eq. (S28) with $j_0 = 0$ and $N = 1$] of the ESEEM of a Bi donor spin coupled to one single ^{29}Si spin with various hyperfine coupling strength. (a,c,e) are the ESEEM and (b,d,f) are the Fourier transform (the ESEEM spectra). The hyperfine coupling is about 2.4 kHz, 13.4 kHz, and 45.5 kHz corresponding to (a/b), (c/d), and (e/f). The external field $B_0 = 1$ G. The microwave pulse is chosen resonant with the transition $6 \leftrightarrow 13$ and of duration 1 μs for the π-pulse.

coupled one, if taken into account, is exactly considered by absorbing it into the hybrid center spin system [corresponding to Eq. (S28) with $j_0 = 1$ and $N = 2$].

Figure S9 shows that while the strongly coupled spin causes some fast, small-amplitude modulations, it has negligible effect on the ESEEM due to the weakly coupled nuclear spin. The weak effect is understandable considering that the matrix elements α_m and α_{m-1} in Eq. (S13) are only slightly ($< 1/10$) modified by the hyperfine coupling to the spin $\mathbf{I}_1$ even when $A_{\text{fc},1}$ is as large as 200 kHz.

Section S6 - Phase cycling scheme

In the tables below is provided the phase cycling scheme for the 3- and 5-pulse ESEEM measurements.

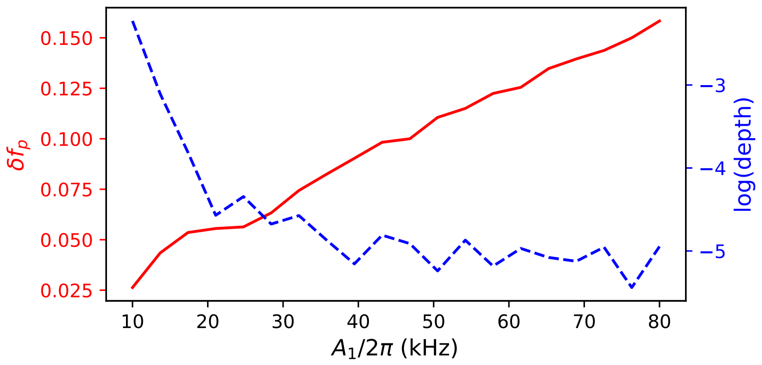

Figure S6. Left axis: The relative frequency deviation ($\delta f_p = |f_{\rm CCE} - f_{\rm Exact}|/f_{\rm Exact}$) of the CCE-1 approximation from the exact results for as a function of the hyperfine coupling A_1 (which refers to Fermi contact coupling in this figure). For CCE-1 to be a good approximation, we can set the cutoff to be 20 kHz, for an error up to 5%. Right axis: The dependence of the modulation depth on A_1. The microwave pulse is chosen resonant with the transition $6 \leftrightarrow 13$ and of duration $1\ \mu$s for the π-pulse. The external field is $B_0 = 1$ G.

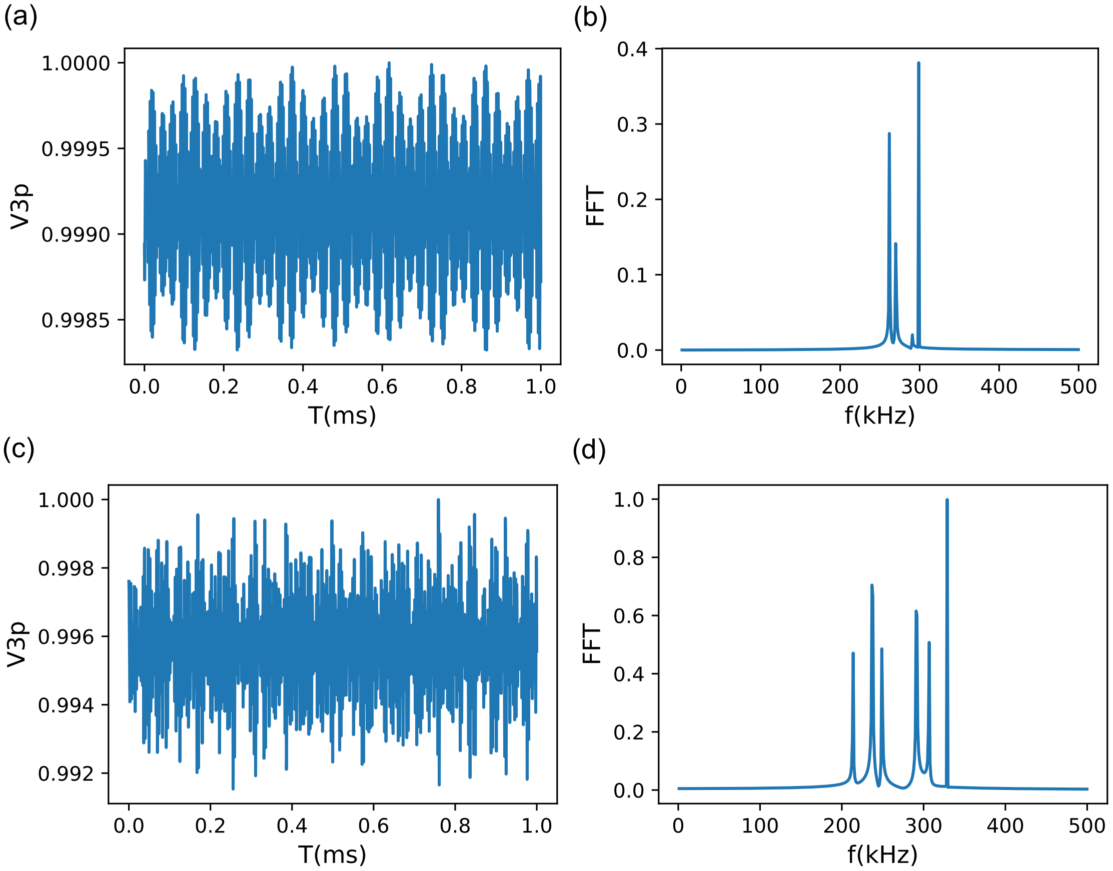

Figure S7. Exact simulation of the 3p-ESEEM due to a strongly coupled ^{29}Si. (a) and (c) are the ESEEM and (b) and (d) are the Fourier transform. The locations and the hyperfine couplings are $\mathbf{r}_1 = (13, 6, -7) \times a_{\rm Si}/4$, $A_{,\rm cf,1} \approx 101.8$ kHz, and $A_{\rm dd,1} \approx 1.6$ kHz for (a) and (b), and $\mathbf{r}_1 = (8, 5, -9) \times a_{\rm Si}/4$, $A_{\rm cf,1} \approx 207.1$ kHz, and $A_{\rm dd,1} \approx 2.8$ kHz for (c) and (d). The external field $B_0 = 1$ G. All pathways with $\left|C^*_{k_0,\mathbf{k}'} C_{k_0,\mathbf{k}}\right| > 10^{-4}$ are taken into account, without imposing the echo condition Eq. (S30).

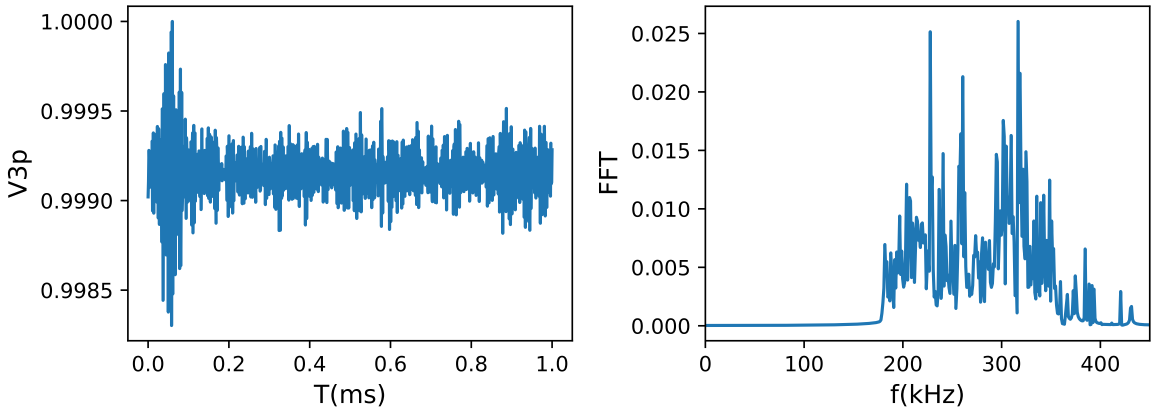

Figure S8. Ensemble average of (a) 3p-ESEEM over 100 samples of nuclear spin states in a thermal distribution and (b) the spectrum. The locations and the hyperfine couplings are $\mathbf{r}_1 = (8, 5, -9) \times a_{\mathrm{Si}}/4$, $A_{\mathrm{cf},1} \approx 207.1$ kHz, and $A_{\mathrm{dd},1} \approx 2.8$ kHz. The external field $B_0 = 1$ G. All pathways with $\left|C^*_{k_0,\mathbf{k}'} C_{k_0,\mathbf{k}}\right| > 10^{-4}$ are taken into account.

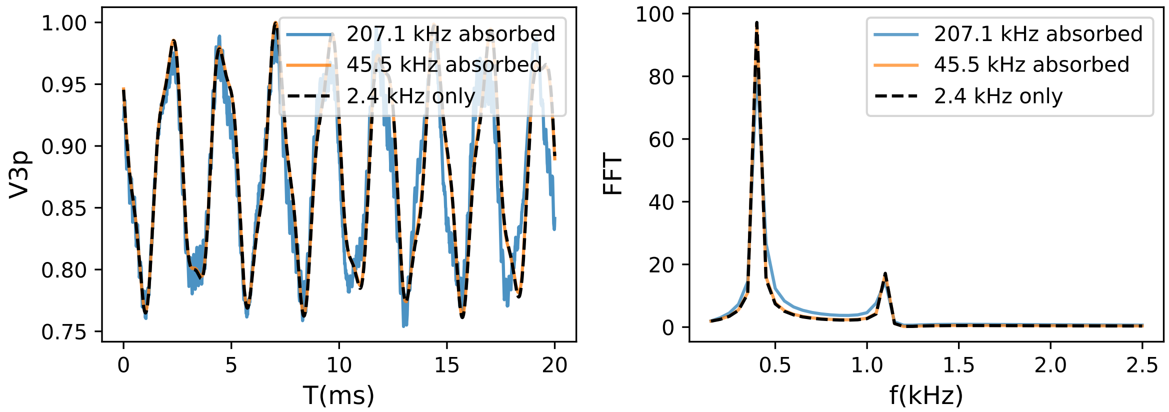

Figure S9. The ESEEM of a weakly coupled ^{29}Si nuclear spin with or without another nuclear spin strongly coupled to the Bi donor spin. The Fermi contact hyperfine coupling $A_{\mathrm{fc},2} \approx 2.4$ kHz for the weakly coupled nuclear spin, and $A_{\mathrm{fc},1} \approx 45.5$ or 207.1 kHz for the strongly coupled nuclear spin. The external field $B_0 = 1$ G.

References

de Sousa, R. and Sarma, S. D.: Theory of nuclear-induced spectral diffusion: Spin decoherence of phosphorus donors in Si and GaAs quantum dots, Physical Review B, 68, https://doi.org/10.1103/physrevb.68.115322, 2003.

Feher, G.: Electron Spin Resonance Experiments on Donors in Silicon. I. Electronic Structure of Donors by the Electron Nuclear Double Resonance Technique, Physical Review, 114, 1219–1244, https://doi.org/10.1103/physrev.114.1219, 1959.

Hale, E. B. and Mieher, R. L.: Shallow Donor Electrons in Silicon. II. Considerations Regarding the Fermi Contact Interactions, Physical Review, 184, 751–759, https://doi.org/10.1103/physrev.184.751, 1969.

Kasumaj, B. and Stoll, S.: 5- and 6-pulse electron spin echo envelope modulation (ESEEM) of multi-nuclear spin systems, Journal of Magnetic Resonance, 190, 233–247, https://doi.org/10.1016/j.jmr.2007.11.001, 2008.

Kohn, W.: Shallow Impurity States in Silicon and Germanium, in: Solid State Physics, pp. 257–320, Elsevier, https://doi.org/10.1016/s0081-1947(08)60104-6, 1957.

Ma, W.-L., Wolfowicz, G., Li, S.-S., Morton, J. J. L., and Liu, R.-B.: Classical nature of nuclear spin noise near clock transitions of Bi donors in silicon, Physical Review B, 92, https://doi.org/10.1103/physrevb.92.161403, 2015.

Yang, W. and Liu, R.-B.: Quantum many-body theory of qubit decoherence in a finite-size spin bath, Physical Review B, 78, https://doi.org/10.1103/PhysRevB.78.085315, 2008.

Table S3. Phase cycling table for 3 pulse ESEEM with CPMG and offset removal.

3 pulse ESEEM				CPMG	
$\pi/2$	$\pi/2$	$\pi/2$	Det.	π	Det.
+x	+x	+x	+y	+y	+y
-x	+x	+x	-y	+y	-y
-x	-x	+x	+y	+y	+y
+x	-x	+x	-y	+y	-y

Table S4. Phase cycling table for 5 pulse ESEEM with CPMG and offset removal.

5 pulse ESEEM						CPMG	
$\pi/2$	π	$\pi/2$	$\pi/2$	π	Det.	π	Det.
+x	+x	+y	+y	+y	+y	+y	+y
-x	-x	-y	+y	+y	+y	+y	+y
-x	-x	-y	-y	-y	-y	+y	-y
+x	+x	+y	-y	-y	-y	+y	-y
+x	-x	-y	-y	+y	+y	+y	+y
-x	+x	+y	-y	+y	+y	+y	+y
-x	+x	+y	+y	-y	-y	+y	-y
+x	-x	-y	+y	-y	-y	+y	-y

Yang, W. and Liu, R.-B.: Quantum many-body theory of qubit decoherence in a finite-size spin bath. II. Ensemble dynamics, Physical Review B, 79, https://doi.org/10.1103/PhysRevB.79.115320, 2009.

Zhao, N., Ho, S.-W., and Liu, R.-B.: Decoherence and dynamical decoupling control of nitrogen vacancy center electron spins in nuclear spin baths, Physical Review B, 85, https://doi.org/10.1103/physrevb.85.115303, 2012.
